# Associations between variants of FADS genes and omega-3 and omega-6 milk fatty acids of Canadian Holstein cows

**DOI:** 10.1186/1471-2156-15-25

**Published:** 2014-02-17

**Authors:** Eveline M Ibeagha-Awemu, Kingsley A Akwanji, Frédéric Beaudoin, Xin Zhao

**Affiliations:** 1 Dairy and Swine Research and Development Centre, Agriculture and Agri-Food Canada, 2000 Rue College, Sherbrooke, Quebec J1M 0C8, Canada; 2 Department of Animal Science, McGill University, 21,111 Lakeshore Road, Ste-Anne-De-Bellevue, Quebec H9X 3V9, Canada

**Keywords:** FADS1, FADS2, SNP, Omega-3 fatty acids, Omega-6 fatty acids, Milk fatty acids, Canadian Holstein cows

## Abstract

**Background:**

Fatty acid desaturase 1 (FADS1) and 2 (FADS2) genes code respectively for the enzymes delta-5 and delta-6 desaturases which are rate limiting enzymes in the synthesis of polyunsaturated omega-3 and omega-6 fatty acids (FAs). Omega-3 and-6 FAs as well as conjugated linoleic acid (CLA) are present in bovine milk and have demonstrated positive health effects in humans. Studies in humans have shown significant relationships between genetic variants in FADS1 and 2 genes with plasma and tissue concentrations of omega-3 and-6 FAs. The aim of this study was to evaluate the extent of sequence variations within these two genes in Canadian Holstein cows as well as the association between sequence variants and health promoting FAs in milk.

**Results:**

Thirty three SNPs were detected within the studied regions of genes including a synonymous mutation (FADS1-07, rs42187261, 306Tyr > Tyr) in exon 8 of FADS1, a non-synonymous mutation (FADS2-14, rs211580559, 294Ala > Val) within FADS2 exon 7, a splice site SNP (FADS2-05, rs211263660), a 3′UTR SNP (FADS2-23, rs109772589), and another 3′UTR SNP with an effect on a microRNA binding site within FADS2 gene (FADS2-19, rs210169303). Association analyses showed significant relations between three out of seven tested SNPs and several FAs. Significant associations (FDR P < 0.05) were recorded between FADS2-23 (rs109772589) and two omega-6 FAs (dihomogamma linolenic acid [C20:3n6] and arachidonic acid [C20:4n6]), FADS1-07 (rs42187261) and one omega-3 FA (eicosapentaenoic acid, C20:5n3) and tricosanoic acid (C23:0), and one intronic SNP, FADS1-01 (rs136261927) and C20:3n6.

**Conclusion:**

Our study has demonstrated positive associations between three SNPs within FADS1 and FADS2 genes (a SNP within the 3’UTR, a synonymous SNP and an intronic SNP), with three milk PUFAs of Canadian Holstein cows thus suggesting possible involvement of synonymous and non-coding region variants in FA synthesis. These SNPs may serve as potential genetic markers in breeding programs to increase milk FAs that are of benefit to human health.

## Background

The polyunsaturated fatty acid (PUFA) concentrations in blood and tissue lipids are known to be closely related to several positive health outcomes on cardiovascular disease morbidity and mortality, early visual, cognitive and motor development, mental health and psychiatric disorders, and early growth and development during pregnancy and early childhood [1]. In addition, PUFAs exert anticancer effects [2] and play beneficial roles in preventing and/or treating various inflammatory immune disorders such as allergies [3,4] and in male fertility and spermatogenesis [5]. Most of these effects are primarily facilitated by the long chain PUFAs (LC-PUFAs) (have 20 carbon atoms or more) arachidonic acid (AA, 20:4n-6), eicosapentaenoic acid (EPA, 20:5n-3) and docosahexaenoic acid (DHA, 22:6n-3). PUFAs are also known to play crucial roles in various cellular functions, such as acting as components of cellular membranes and as precursors of important mediators of inflammation (e.g. EPA, [6]). PUFAs of 18 carbon atoms are mainly composed of isomers of conjugated linoleic acid (CLA). They too like LC-PUFAs have demonstrated health related benefits such as antiadipogenic, anticarcinogenic, antiatherogenic, antidiabetogenic and antiinflammatory properties (review by [7,8]). LC-PUFAs are classified in three principal families, omega-6 (n-6), omega-3 (n-3) or omega-9 (n-9) fatty acids (FAs), according to the position of the terminal double bond from the methyl end of the FA [9]. The precursor FAs of omega-3 and -6 families are respectively linoleic acid (LA) and alpha linolenic acid (ALA). They cannot be synthesised by mammals so they must be provided by the diet and are therefore defined as essential FAs. Sources of unsaturated fatty acids (USFAs) including monounsaturated fatty acids (MUFAs) and PUFAs in human diets are plant oils, oil seeds, and animal products including fish, meat, eggs and milk. The major source of CLA is from ruminant products, mainly milk and dairy products. Rumenic acid is the major form of CLA in milk and it is endogenously synthesized through the activity of stearoyl-CoA desaturase 1 (SCD1) enzyme or delta 9 desaturase. SCD1 desaturates vaccenic acid, an intermediate in rumen biohydrogenation to form CLA [10]. Mammals or humans can also synthesize LC-PUFAs endogenously starting from the precursor essential FAs, LA and ALA. In western diets, bovine milk is therefore a major contributor of CLA, omega-3 and omega-6 FAs to humans. However, the proportion of these beneficial FAs in bovine milk is generally low.

Endogenous synthesis of omega-3 and -6 FAs is through desaturation of LA and ALA by the activities of fatty acid synthase 1 (FADS1) and 2 (FADS2), and elongases. FADS1 and FADS2 code, respectively, for the enzymes Δ-5 and Δ-6 desaturases considered rate limiting enzymes in the synthesis of LC-PUFAs [11,12]. FADS1 and FADS2 add double bonds at the Δ-5 and Δ-6 position, respectively, of LC-PUFAs. These genes are clustered in a head-on-head direction on bovine chromosome 29 (BTA 29). A third desaturase gene, FADS3 is also clustered at the same location but its role in FA metabolism in cows is not yet clear. The concentration of PUFAs in bovine milk is known to be influenced by dietary factors [13,14] and genetics/breed [15-17]. Furthermore, low to medium heritability values for USFAs recorded for many breeds [18,19] indicate the possibility of improvement through genetic selection. In humans, several studies suggest that plasma and tissue concentrations of omega-3 and -6 FAs are associated with several single nucleotide polymorphisms (SNPs) in the FADS1 and FADS2 genes [20-23]. Also, recent gene and genome-wide association studies (GWAS) in humans have highlighted the influence of variations in the FADS1 and FADS2 gene cluster on lipid metabolism, glucose metabolism, and other quantitative traits such as total cholesterol and low-density lipoprotein [24-26], and disease conditions such as development of metabolic syndrome [27], coronary artery disease [28], myocardial infarction [29], and dyslipidemia [30]. In these studies, changes in the levels of individual FAs related to FADS enzyme activity were noted, thus suggesting a relationship between these conditions and a deregulation in desaturase activity. In a recent study, Merino et al. [31] demonstrated that genetic variants in FADS1 and FADS2 could alter desaturase activities in subjects of Caucasians and Asian descent. Even though several sequence variations including SNPs are listed for these genes for bovine in the SNP database (dbSNP, http://www.ncbi.nlm.nih.gov/projects/SNP/), no association with milk FAs contents has been attempted. Increasing the proportion of USFAs, especially PUFAs in milk may greatly influence human health and wellness. Dietary manipulations through feeding diets rich in USFAs have proven successful but come with significant extra cost. Exploiting animal’s inherent abilities through searching for FADS gene variants with increased desaturase activities and including them in breeding programs may lead to increased milk PUFA content.

The aim of this study was to characterize the FADS1 and FADS2 genes of Canadian Holstein cows for sequence variations and to determine possible associations with beneficial FAs content in milk.

## Methods

### Animal ethics

Animal care use and protocols were approved by the McGill University Animal Care Committee.

### Screening for polymorphisms in FADS genes

DNA from 40 randomly selected Holstein cows from the Macdonald Campus Dairy Unit, McGill University were used to screen for polymorphisms within the FADS genes. DNA was isolated from leucocytes using a modified method of Montgomery and Sise [32]. To screen for polymorphisms, only the regulatory regions (promoters, 5′ and 3′UTRs) and coding regions (exons including about 200 bp of surrounding intronic sequences) were targeted for selective amplification by PCR and Sanger sequencing. Primers targeting the regions of interest were designed using primer3 program and the reference sequences NC_007330.4 (region 41911653-41925160, FADS1) and NC_007330.4 (region 41998252-42036271, FADS2). After optimization, each PCR reaction was carried out in a final volume of 40 μL. PCR reaction mix contained 30 ng of genomic DNA, 0.25 mM dNTPs, 1.5 to 3.0 mM MgCl2 (Additional file 1: Table S1), 10 μM each primer, 1× Crimpson *Taq* buffer and 2 units Crimpson Tag DNA polymerase (NEB Canada, Pickering, ON, Canada). PCR cycling conditions with an Eppendorf Mastercycler® pro (Eppendorf North America, Hauppauge, NY, USA) included an initial denaturation step at 94°C for 2 min followed by 30 cycles of 94°C for 30 sec, 56 to 62°C (Additional file 1: Table S1) for 30 sec and 72°C for 1 min, plus a final elongation step of 72°C for 5 min. Successful PCR amplification was confirmed by running 5 μL of PCR products on 1% agarose gels and visualization under UV rays. Thirty μL of PCR products were sent to McGill University/Genome Quebec Innovation Centre for Sanger sequencing using the big dye termination technique and an ABI 3700 sequencer. Sequences were processed for polymorphisms with ChromasPro version 1.7.3 (http://technelysium.com.au/) and ABI Prism SeqScape® version 2.1 (Life Technologies Inc., Burlington, ON, Canada) software. Effects of regulatory region polymorphisms on microRNA binding sites were searched with TargetScan Release 6.0 (http://www.targetscan.org/vert_60/).

### Milk sampling and fatty acid analysis

Milk samples were collected from 450 cows from five herds in the province of Quebec. Cows were in mid-lactation and their parities ranged from 1 to 4. Milk sample collection was coordinated by Valacta, the dairy production center of expertise for Quebec and the Atlantic provinces (http://www.valacta.com). Milk samples were centrifuged (12000 × g at 4°C for 30 min) immediately upon arrival at the laboratory to separate the fat from the whey and somatic cell fractions. The fat portion was used for FA analysis while the somatic cells were used for DNA isolation. The different FA isomers were separated by capillary gas chromatography on a Varian CP-3900 gas chromatograph equipped with a Varian CP-8400 auto-sampler and auto-injector, column oven and a flame ionization detector (Varian Inc., Walnut Creek, CA, USA) according to O’Fallon et al. [33].

### Genomic DNA purification from milk somatic cells

Genomic DNA was isolated from milk somatic cells using NucleoSpin® Blood QuickPure kit (MJS Biolynx, Ontario, Canada) with some modifications. Briefly, somatic cells were washed twice with phosphate buffered saline (PBS) and resuspended in 200 µL of TE buffer (10 mMTris-HCl and 1 mM EDTA pH 7.6). Three hundred µL of 0.5 M EDTA was added and mixed by shaking vigorously for 45 min followed by centrifugation for 10 min at 3000 × g. The pellet was resuspended in 200 µL of PBS. Twenty five μL proteinase K solution (10 mg/mL) and 200 µL of lysis buffer BQ1 (NucleoSpin® kit) were added to each tube before incubation for 15 min at 70°C. Seven hundred µL of chloroform (Sigma, Ontario, Canada) was added and tubes were vigorously shaken (15 secs). After 10 min of centrifugation at 14000 × g, the aqueous phase was transferred to a 1.5 mL tube containing 210 µL of anhydrous ethanol, mixed briefly and loaded on the NucleoSpin® blood column. The standard protocol of the NucleoSpin® kit was followed from this point except that three washes were done instead of two. Genomic DNA concentration was measured with a NanoDrop® spectrophotometer (NanoDrop Technologies, Inc., Wilmington, DE, USA).

### SNP genotyping

Five potentially functional SNPs (includes one synonymous, one non-synonymous, one splice site and two 3′UTR mutations) and two randomly selected intronic mutations within FADS1 and FADS2 genes (Table 1), out of 33 detected in this study were genotyped in 450 samples and used in association analyses. Genotyping was accomplished with TaqMan probes and a StepOnePlus™ Real-Time PCR system (Applied Biosystems, Foster City, CA, USA). All reagents required for the TaqMan assay including universal master mix, amplifying primers and probes were obtained from Applied Biosystems. One allelic probe was labeled with FAM dye and the other with the fluorescent VIC dye (Additional file 2: Table S2). PCR was run in the TaqMan universal master mix at a probe concentration of 200 nM. The reaction was performed in a 96-well format in a total reaction volume of 10 µL using 10 ng of gDNA. The real-time PCR cycling condition was as follows: reaction plates were heated for 30 secs at 60°C and then for 10 minutes at 95°C, followed by 35 cycles of 92°C for 15 secs and 60°C for 1 minute. The fluorescence intensity of each well in the TaqMan assay plate was subsequently read and the SNP call from the system (StepOne Software v2.2.2) was used to determine the genotype of each animal.

**Table 1:** Genotyped markers, frequencies of alleles and genotypes, and test for Hardy-Weinberg equilibrium

**Marker**^**1**^		**Location/characteristic of SNP**	**Allele**	**Count**	**Frequency**	**Genotype**	**Count**	**Frequency**	**Test for HWE**
**Lab Id**	**rs#**								**P-values**
FADS1-01	rs136261927	Intron	C	685	0.812	CC	275	0.652	0.4181
			T	159	0.188	CT	135	0.320	
						TT	12	0.028	
FADS1-07	rs42187261	Exon, synonymous (306Tyr > Tyr)	C	349	0.428	CC	70	0.172	0.3695
			T	467	0.572	CT	209	0.512	
						TT	129	0.316	
FADS1-08	rs41652284	Intron	C	626	0.742	CC	226	0.536	0.1289
			T	218	0.258	CT	174	0.412	
						TT	22	0.052	
FADS2-05	rs211263660	Intron (splice site)	C	523	0.612	CC	163	0.382	0.6135
			T	331	0.388	CT	197	0.461	
						TT	67	0.157	
FADS2-14	rs211580559	Exon, non synonymous (294 Ala > Val)	C	517	0.597	CC	159	0.367	0.3746
			T	349	0.403	CT	199	0.460	
						TT	75	0.173	
FADS2-19	rs210169303	3′UTR (microRNA binding site)	C	589	0.731	CC	219	0.543	0.3764
			T	217	0.269	CT	151	0.375	
						TT	33	0.082	
FADS2-23	rs109772589	3′UTR	A	673	0.807	AA	267	0.640	0.2099
			G	161	0.193	AG	139	0.333	
						GG	11	0.026	

^1^Lab Id = lab notation of SNPs, rs# = reference SNP ID number.

### Statistical analyses

Statistical analyses were performed with SAS version 9.3 software (SAS Institute Inc., Cary, NC, USA). ALLELE procedure was used to compute allele frequencies, genotype frequencies and tests for HWE employing Fisher’s exact test. Associations between genotypes and the different FA profiles were evaluated using PROC MIXED with genotype as fixed effect (model 1).

Model 1: Y_ijk_ = μ + H_i_ + G_j_ + e_ijk_

Where Y_ijk_ = trait measured; μ = overall mean; H_i_ = fixed effect of herd; G_j_ = fixed effect of genotype (j = 3, each SNP was analyzed separately with the same model) and e_ijk_ = residual error. A false discovery rate (FDR) was applied on the raw p-values of the global genotype effect using the MULTTEST procedure of SAS. For the FAs with significant results, least square means multiple comparisons were done with Tukey adjustments and considered to be significantly different at P ≤0.05. Spearman correlations between FAs were calculated over all data with the CORR procedure of SAS.

Allele substitution effects were estimated using PROC MIXED and the following model (Model 2).

Model 2: Y_ij_ = μ + H_i_ + ASE + e_ij_

Where Y_ij_ = trait measured; μ = overall mean; H_i_ = fixed effect of herd; ASE = allele substitution effects (alleles at the same locus were coded 0, 1 or 2 [where 0 = CC or GG, 1 = CT or AG and 2 = TT or AA]) and treated as a linear covariant and e_ij_ = residual error. FDR was also applied on the raw p-values of the estimates.

## Results

### SNPs within the coding and regulatory regions of FADS1 and FADS2 genes in Canadian Holstein cows

A total of 33 SNPs were detected within the studied regions, 9 within FADS1 and 24 within FADS2, in Canadian Holstein cows (Additional file 3: Table S3).The SNPs have been submitted to the Single Nucleotide Polymorphism Database (dbSNP). Two SNPs are located within the coding regions, a synonymous mutation (FADS1-07, rs42187261) in exon 8 of FADS1 with no effect on amino acid 306 (306Tyr > Tyr) and a non-synonymous mutation (FADS2-14, rs211580559) within FADS2 exon 7 that causes a change in amino acid 294 from alanine to valine (294Ala > Val). One SNP (FADS2-05, rs211263660) occurred within a splice site. Seven SNPs were identified within the 3′UTR of FADS2 gene and Target Scan analysis indicated that FADS2-19 (rs210169303) is situated within the binding site for bta-miR-744. The presence of the mutated allele (T) may disrupt binding of this microRNA. Frequencies of genotyped alleles are shown in Table 1 and are all in agreement with Hardy-Weinberg equilibrium. The distribution of genotypes and their frequencies are shown in Table 1.

### FADS SNP association with milk polyunsaturated fatty acids (PUFAs)

Results of significant SNP marker associations with milk PUFAs are shown in Table 2, while allele substitution results are presented in Table 3. Complete results, including significant and non-significant associations as well as substitution effects are shown in Additional file 4: Table S4 and Additional file 5: Table S5. Our results indicate that three markers (FADS1-01, FADS1-07 and FADS2-23) were significantly associated with three individual milk PUFAs (dihomogamma linolenic acid [C20:3n6], arachidonic acid [C20:4n6] and eicosapentaenoic acid [C20:5n3]) (FDR P < 0.05, Table 2). In addition, there was a significant association between FADS1-07 and tricosanoic acid (C23:0), a saturated FA. Out of these associations, significant relationship between FADS1-07, a synonymous mutation within FADS1 gene, and one omega-3 FA (C20:5n3, eicosapentanoic acid) showed that genotype TT was linked to the highest increase in eicosapentanoic acid. Allele substitution at this locus (allele T substituted for C) indicated that allele T increased milk C20:5n3 by 0.00420 ± 0.0008 (Table 3). A significant association was also recorded between one of the evaluated intronic mutations within FADS1 gene (FADS1-01[rs136261927]) and the omega-6 FA, C20:3n6. In this case, genotype CC was associated with the highest increase in milk C20:3n6 content. This result is supported by allele substitution analysis whereby substituting allele T for C at FADS1-01 associated a decrease of -0.009 ± 0.002 of milk C20:3n6 content to allele T (Table 3). FADS2-23, a 3′UTR variant, was significantly associated with the omega-6 FAs, C20:3n6 and C20:4n6, with genotype GG showing higher increases in the affected FAs (Table 2). Similarly, allele substitution effects shows that allele A at this locus decreased milk C20:3n6 and C20:4n6 significantly (Table 3).

**Table 2:** **Results of significant**^**1 **^**marker associations with milk fatty acid isomers (g/100 g of total fat) of Canadian Holstein cows**

**Trait**		**SNP**^**2**^		**Means of genotypes**^**3**^						**Max SEM**^**4**^	**P-value**	**FDR**^**5**^
**Fatty acid**	**Common name**	**Lab Id**	**rs#**	**CC**	**CT**	**TT**	**AA**	**AG**	**GG**			
												**P-value**
**Polyunsaturated fatty acids**												
C18:2n10t12c	CLA: 10t12c	FADS2-19	rs210169303	0.024^a^	0.023^ab^	0.021^b^	-	-	-	0.001	0.027	0.189
C18:2n6tt	Trans linoleic acid	FADS1-07	rs42187261	0.158^ab^	0.157^a^	0.170^b^	-	-	-	0.006	0.034	0.176
		FADS2-19	rs210169303	0.165^a^	0.158^ab^	0.147^b^	-	-	-	0.008	0.050	0.176
C20:3n6	Dihomogamma linolenic acid	FADS1-01	rs136261927	0.095^a^	0.087^b^	0.075^b^	-	-	-	0.007	0.0003	0.002
		FADS1-07	rs42187261	0.090^ab^	0.089^a^	0.096^b^	-	-	-	0.003	0.024	0.056
		FADS2-23	rs109772589	-	-	-	0.089^a^	0.096^b^	0.108^b^	0.007	0.001	0.003
C20:4n6	Arachidonic acid	FADS1-07	rs42187261	0.114^a^	0.116^a^	0.122^b^	-	-	-	0.003	0.021	0.074
		FADS2-23	rs109772589	-	-	-	0.115^a^	0.123^b^	0.126^ab^	0.007	0.002	0.013
C20:5n3	Eicosapentaenoic acid	FADS1-07	rs42187261	0.036^ab^	0.033^a^	0.037^b^	-	-	-	0.001	0.004	0.029
**Monounsaturated fatty acids**												
C14:1 t	Myristelaidic acid	FADS2-23	rs109772589	-	-	-	0.247^ab^	0.259^a^	0.213^b^	0.019	0.029	0.204
C18:1 total		FADS1-07	rs42187261	24.458^a^	24.439^a^	23.106^b^	-	-	-	0.544	0.012	0.084
C18:1n9c	Oleic acid	FADS1-07	rs42187261	21.419^a^	21.374^a^	20.089^b^	-	-	-	0.520	0.011	0.074
Total MUFA		FADS1-07	rs42187261	30.598^a^	30.669^a^	29.207^b^	-	-	-	0.620	0.019	0.131
**Saturated fatty acids**												
C8:0	Caprylic acid	FADS1-07	rs42187261	0.876^a^	0.883^a^	0.921^b^	-	-	-	0.020	0.043	0.301
C11:0	Undecanoic acid	FADS1-07	rs42187261	0.217^a^	0.221^a^	0.238^b^	-	-	-	0.007	0.017	0.116
C15:0	Pentadecylic acid	FADS1-08	rs41652284	1.147^a^	1.098^a^	1.262^b^	-	-	-	0.058	0.010	0.072
C17:0	Margaric acid	FADS1-08	rs41652284	0.669^ab^	0.658^a^	0.698^b^	-	-	-	0.016	0.031	0.219
C23:0	Tricosanoic acid	FADS1-07	rs42187261	0.024^a^	0.023^a^	0.028^b^	-	-	-	0.001	0.002	0.011
Total SFA		FADS1-07	rs42187261	66.155^ab^	66.051^a^	67.542^b^	-	-	-	0.638	0.021	0.147

^1^For significant associations, P-values and FDR P-values (P≤0.05) are shown.

^2^Lab Id = lab notation of SNPs, rs# = SNP data base identification number of SNPs.

^3^For each trait, genotype means (g/100 g of total fat) with different superscripts differ significantly (P ≤ 0.05). Genotype frequencies are listed in Table 2.

^4^Maximum standard error of the mean.

^5^FDR = False discovery rate.

**Table 3:** **Results of significant**^**1 **^**allele substitution effects P-value**

**Trait**		**SNP**^**2**^		**Estimate ± SE**^**3**^	**P-values**	**FDR**^**4**^
**Fatty acid**	**Common name**	**Lab Id**	**rs#**			**P-values**
**Polyunsaturated fatty acids**						
C18:2n9c11t	CLA:9c11t	FADS2-19	rs210169303	-0.0176 ± 0.0083	0.036	0.248
C18:2n10t12c	CLA:10t12c	FADS2-19	rs210169303	-0.0012 ± 0.0005	0.010	0.069
C18:2n6cc	Linoleic acid	FADS1-07	rs42187261	-0.0410 ± 0.0197	0.038	0.137
		FADS1-08	rs41652284	-0.0468 ± 0.0226	0.039	0.137
C18:2n6tt	Trans linoleic acid	FADS1-07	rs42187261	0.0069 ± 0.0033	0.036	0.126
		FADS2-19	rs210169303	-0.0083 ± 0.0034	0.016	0.109
C20:3n6	Dihomogamma linolenic acid	FADS1-01	rs136261927	-0.0087 ± 0.0021	0.000	0.0004
		FADS1-07	rs42187261	0.0037 ± 0.0017	0.032	0.055
		FADS1-08	rs41652284	-0.0044 ± 0.0020	0.027	0.055
		FADS2-23	rs109772589	-0.0080 ± 0.0021	0.0002	0.001
C20:4n6	Arachidonic acid	FADS1-07	rs42187261	0.0042 ± 0.0016	0.009	0.032
		FADS2-23	rs109772589	-0.0071 ± 0.0020	0.0004	0.003
C20:5n3	Eicosapentaenoic acid	FADS1-07	rs42187261	0.00420 ± 0.0008	0.0036	0.039
**Monounsaturated fatty acids**						
C14:1 t	Myristelaidic acid	FADS2-05	rs211263660	0.0092 ± 0.0046	0.047	0.256
C18:1n9c	Oleic acid	FADS1-07	rs42187261	-0.7529 ± 0.2889	0.009	0.067
C18:1 total		FADS1-07	rs42187261	-0.7697 ± 0.3023	0.011	0.079
Total MUFA		FADS1-07	rs42187261	-0.8044 ± 0.3445	0.020	0.141
**Saturated fatty acids**						
C6:0	Caproic acid	FADS1-07	rs42187261	0.0215 ± 0.0110	0.050	0.353
C8:0	Caprylic acid	FADS1-07	rs42187261	0.0246 ± 0.0108	0.023	0.158
C10:0	Caprinic acid	FADS1-07	rs42187261	0.087 2 ± 0.0382	0.023	0.161
C11:0	Undecanoic acid	FADS1-07	rs42187261	0.0111 ± 0.0041	0.008	0.053
C12:0	Lauric acid	FADS1-07	rs42187261	0.1249 ± 0.0525	0.018	0.125
C16:0	Palmitic acid	FADS1-01	rs136261927	0.6519 ± 0.3287	0.048	0.084
		FADS1-07	rs42187261	0.5063 ± 0.2555	0.048	0.084
		FADS2-05	rs209202414	-0.5908 ± 0.2473	0.017	0.079
		FADS2-19	rs210169303	0.6281 ± 0.2746	0.023	0.079
C23:0	Tricosanoic acid	FADS1-07	rs42187261	0.0021 ± 0.0008	0.006	0.044
Total SFA		FADS1-07	rs42187261	0.8069 ± 0.3549	0.024	0.165

^1^For significant associations, P-values and FDR P-values (P≤0.05) are shown. Reference genotype is either CC or GG.

^2^Lab Id = lab notation of SNPs, rs# = SNP data base identification number of SNPs.

^3^Estimate = g/100 g of total fat, SE = Standard error.

^4^FDR = False discovery rate.

### FADS SNP association with milk monounsaturated fatty acids (MUFAs)

Significant associations (raw p-values) recorded between two studied SNPs and two MUFA isomers as well as total MUFA (Tables 2 and 3) disappeared after FDR correction. Complete association results and allele substitution effects are shown in Additional file 4: Table S4 and Additional file 5: Table S5, respectively.

### FADS SNP association with milk saturated fatty acids (SFAs)

Only one significant association was recorded between FADS1-07 and one saturated FA (C23:0, tracosanoic acid). Genotype TT was linked to the highest increases in tracosanoic acid. Allele substitution at this locus (allele T substituted for C) indicated that allele T increased milk C23:0 by 0.0021 ± 0.0008. Complete association results and allele substitution effects are shown in Tables 2 and 3, Additional file 4: Table S4 and Additional file 5: Table S5, respectively.

### Correlations between fatty acids

Correlations between total SFAs and total MUFAs was positive but very low (0.099), and between total SFAs and total PUFAs was moderate but negative (-0.343) (Table 4). SFAs of 10 to 16 chain lengths showed very high but negative correlations with total MUFAs (-0.903) and low but also negative with total PUFAs (-0.268). Between total MUFAs and total PUFAs, results showed a low but positive relationship (0.234). Details of Pearson correlation coefficients between individual FAs are shown in Additional file 6: Table S6.

**Table 4:** Pearson correlation coefficients between total PUFAs, MUFAs, SFAs and different SFA classes

	**PUFA**	**MUFA**	**SFA**	**C4:0 to C8:0**	**C10:0 to C16:0**	**C18:0 to C24:0**
PUFA	1.000	0.234	-0.343	0.026	-0.268	0.004
MUFA	0.234	1.000	0.099	-0.402	-0.903	0.386
SFA	-0.343	0.099	1.000	0.391	0.904	-0.375
C4:0 to C8:0	0.026	-0.402	0.391	1.000	0.175	0.139
C10:0 to C16:0	-0.268	-0.903	0.904	0.175	1.000	-0.698
C18:0 to C24:0	0.004	0.386	-0.375	0.139	-0.698	1.000

## Discussion

Our study is the first to report SNP characterization within bovine FADS1 and FADS2 genes in Canadian Holstein cows, and association with milk SFAs, MUFAs and PUFAs. FADS1 and FADS2 genes code for Δ-5 and Δ-6 desaturases, respectively and these enzymes are crucial in the endogenous synthesis of LC-PUFAs from the precursor essential FAs, LA and ALA, obtained from the diet. Numerous human studies have indicated that, changes in the levels of individual FAs are related to FADS enzyme activity [27-30] and recently, Merino et al [31] reported that genetic variants in FADS1 and FADS2 genes in humans can alter desaturase activity. Furthermore, it has been shown in humans that genetic variants of FADS1 and FADS2 influence blood lipid and breast milk essential FAs in pregnancy and lactation [34]. In this study, mutations were detected in the coding as well as non-coding (introns and 3′UTR) regions of studied genes. Mutations in these regions have been variously shown to associate significantly with economically important traits in farm animals, affect gene expression or function of resultant protein products [35,36].

The reported non-synonymous mutation within FADS2 gene has either valine or alanine at position 294 of the protein. These two amino acids are both nonpolar amino acids with aliphatic side chain groups. This change in amino acid does not seem to affect the structure and function of the resultant protein which may explain why no significant association was recorded between this SNP and any of studied FAs.

Significant associations were recorded between one PUFA (C20:5n3), and the synonymous FADS1 (FADS1-07, rs42187261) mutation. According to allele substitution results, allele T, the major allele with frequency of 57% in studied individuals, was superior in increasing the content of C20:5n3. It is worthwhile to point out that positive influence of this SNP on oleic acid and other health promoting FAs was significant before FDR correction, but not after FDR correction. Stearic acid (C18:0), a SFA and one of the main products of extensive biohydrogenation of USFAs in the ruminal environment is desaturated by the SCD1 enzyme [37] to oleic acid. The influence of this SNP (FADS1-07, rs42187261), together with favorable associations with SNPs detected within the SCD1 gene [38-40] may contribute to desaturate products of ruminal biohydrogenation or SFAs to much needed health promoting FAs. Furthermore, this mutation (rs42187261) also favored increase in the content of one individual SFA (C23:0). This study shows only a moderate correlation between C23:0 and C20:5n3 (r^2^ = 0.459). Furthermore, C23:0 is not among SFAs (C16:0, C14:0 and C12:0) which were associated with raised plasma low density lipoprotein cholesterol levels [41]. Since most SFAs in milk are synthesized de novo in the mammary gland through the activities of enzymes other than FADS1 and FADS2 while USFAs arise mostly from dietary sources, the effect of this mutation (rs42187261) may be more relevant for USFAs. Furthermore, very low and in most cases negative correlations were recorded between PUFAs positively influenced in this study and SFAs; implying that the effect of the rs42187261, rs109772589 and rs136261927 mutations can be useful in the positive management of these PUFAs.

Synonymous mutations have generally been regarded as silent mutations, that is, have no effect on protein structure and function. Recent evidence, however, indicates that silent mutations are able to cause changes in protein expression, conformation and function [36]. Numerous recent GWAS studies have revealed substantial contributions of synonymous SNPs to human disease risk and other complex traits [42-45]. Evidence suggest that synonymous SNPs can result in aberrant mRNA splicing [46], affect mRNA stability and thus protein expression and enzymatic activity [44], affect protein conformation and have functional and clinical consequences [47]. Consequently, Chamary and Hurst [48] estimated that 5-10% of human genes contain at least one region in which silent mutations could be harmful. Synonymous SNPs are generally not included in most association studies between SNPs and milk fat traits in ruminants. Therefore, important information that can assist in breeding programs could be missed. Our result on the synonymous FADS1 (FADS1-07, rs42187261) mutation support the concept of using both non-synonymous and synonymous mutations in animal breeding.

Our study also recorded a significant association between one intronic SNP (FADS1-01, rs136261927) and one MUFA (C20:3n6). Reports of significant associations between intronic SNPs in several lipogenic genes and milk fat traits as well as other production traits have emerged [35]. For example, intronic SNPs in the SCD1 (g.6926A > G) and FASN genes (g.8948C > T, ss491228481) were recently shown to be significantly associated with milk yield, fat yield and protein yield in Chinese Holstein cows [49]. Mutations in intronic regions are known to play important regulatory roles in mammalian gene expression, including roles in transcription regulation, polyadenylation, mRNA export, translational efficiency, and the rate of mRNA decay [50]. Our study further points to the importance of non-coding region SNPs in the modulation of gene expression and resultant effects on milk yield traits.

## Conclusion

Our study has uncovered positive associations between three out of seven tested SNPs and health promoting FAs in the milk of Canadian Holstein cows. These SNPs may serve as potential genetic markers that can be used in breeding programs for increased milk beneficial FAs.

## Competing interests

The authors have no competing interests to declare.

## Authors’ contributions

EMIA and XZ designed the study and wrote the paper. KAA isolated DNA from milk somatic cells, prepared samples for Sanger sequencing and performed gas chromatographic analysis of milk fatty acids, FB performed SNP genotyping and contributed to manuscript writing. All authors read and approved the final manuscript.

## Supplementary Material

Additional file 1: Table S1 Primer sequences used in charactering FADS genes by Sanger sequencing. Reference sequences used in primer design with primer3 program were NC_007330.4 (region 41911653-41925160, FADS1) and NC_007330.4 (region 41998252-42036271, FADS2). Click here for file

Additional file 2: Table S2 Primers and probes used in the TaqMan assay for SNP genotyping. Click here for file

Additional file 3: Table S3 Identified SNPs within studied regions of FADS1 and FADS2 genes and functional annotation. Click here for file

Additional file 4: Table S4 Complete marker-trait association results. Significant P-values (P ≤ 0.05) are highlighted yellow. Click here for file

Additional file 5: Table S5 Complete results on allele substitution effect. The reference genotype is either CC or GG. Significant p-values (P ≤ 0.05) are highlighted yellow. Click here for file

Additional file 6: Table S6 Pearson correlation coefficients between studied fatty acids. Click here for file
