# Supplementary material for: Associations between variants of FADS genes and omega-3 and omega-6 milk fatty acids of Canadian Holstein cows
Source: BMC Genet. 2014 Feb 17;15:25. doi: 10.1186/1471-2156-15-25 (PMC3929906; doi:10.1186/1471-2156-15-25)
Supplement: Additional file 1: Table S1 — Primer sequences used in charactering FADS genes by Sanger sequencing. Reference sequences used in primer design with primer3 program were NC_007330.4 (region 41911653-41925160, FADS1) and NC_007330.4 (region 41998252-42036271, FADS2). [file 1471-2156-15-25-S1.docx]

Table S1: Primer sequences used in characterizing FADS genes by Sanger sequencing. Reference sequences used in primer design with primer3 program were NC_007330.4 (region 41911653-41925160, FADS1) and NC_007330.4 (region 41998252-42036271, FADS2)

| **Primer Name^1^** | | **Primer Sequence (5’ to 3’)** | **Length bp** | **AT^2^/MgCl2 concentration** | **Sequencing primer** |
| --- | --- | --- | --- | --- | --- |
| **FADS1 Gene** | |  |  |  |  |
| FADS1Prom_14F | | AGC TCC AAG CCC CTA AGC TA | 715 | 60°C, 1.5 | FADS1Prom_728R |
| FADS1Prom_728R | | GGA CAA CGG TTC CTG TGA AG |  |  |  |
| FADS1Ex1_754F | | AAG GCG GGA GTC GAA AAG T | 717 | 60°C, 3.0 | FADS1Ex1_754F |
| FADS1Ex1_1470R | | CCA GTT ACC CAG CAA CAG GT |  |  |  |
| FADS1Ex2_2371F | | TCA GCC TTC TTC ACA GTC CA | 712 | 60°C, 2.0 | FADS1Ex2_2371F |
| FADS1Ex2_3082R | | TCT ACC CTT GCT CTG CCA CT |  |  |  |
| FADS1Ex3_3954F | | GAG CAC TTA ACT CTG TGC CAA G | 805 | 60 °C, 2.0 | FADS1Ex3_3954F |
| FADS1Ex3_4758R | | TGG TTT TTG ACA CAC CTG GA |  |  |  |
| FADS1Ex4/5_4867F | | GTT TGT CAC TCC GTC CCA GT | 888 | 60°C, 1.5 | FADS1Ex4/5_4867F |
| FADS1Ex4/5_5754R | | CAG CAT TTG CAC AGC AAT CT |  |  |  |
| FADS1 Ex6_8468F | | GAG GCC CTG GAA ACC TTT A | 648 | 60°C, 2.0 | FADS1 Ex6_8468F |
| FADS1 Ex6_9115R | | CTC CCC ACC ACT CAG TTC C |  |  |  |
| FADS1Ex7_10412F | | CAG CTT CCA CAC CAA GTG AA | 667 | 60°C, 1.5 | FADS1Ex7_10412F |
| FADS1Ex7_11078R | | AGT AGG GGC CGT CTC TCA TT |  |  |  |
| FADS1Ex8/9_11384F | | TGT GGT CCT GGA AGA TTC GT | 790 | 60°C, 2.0 | FADS1Ex8/9_11384F |
| FADS1Ex8/9_12173R | | CAC TCC CTT CTC CAC ATG GT |  |  |  |
| FADS1Ex10/11_12068F | | TGA AAG ACC TGG GCA TAA CA | 809 | 60°C, 1.5 | FADS1Ex10/11_12068F |
| FADS1Ex10/11_12876R | | AGG TGG CTG TTA TTG GTG GA |  |  |  |
| FADS1Ex12_13350F | | GAA TTC TGA GGC CTG GCA TA | 1183 | 58°C, 1.8 | FADS1Ex12_13350F |
| FADS1Ex12_14532R | | CCT GGT TTC CTC TCT GTG TG |  |  |  |
| **FADS2 gene** |  | |  |  |  |
| FADS2Ex1_944F | GGA GAA GAC AAA AGC CGA AAG | | 611 | 60°C, 2.0 | FADS2 Ex1_944F |
| FADS2Ex1_1554R | GTC TCG GGC TAC GGA TGA AC | |  |  |  |
| FADS2Ex2_11621F | AGG CTC CAG GCT TGT TCT TT | | 716 | 60°C, 3.0 | FADS2__Ex2_11621F |
| FADS2Ex2_12336R | CAA ACC CAA GCT GGC ACA | |  |  |  |
| FADS2Ex3/4_13392F | ACA CGG AAT GGA CAG CAT TT | | 922 | 60°C, 2.0 | FADS2Ex3/4_13392F |
| FADS2Ex3/4_14313R | TAC TGG CCC CAA ATT GAA AG | |  |  |  |
| FADS2Ex5_19345F | TGG AAG GGT GGA GTA AGC | | 687 | 60°C, 2.0 | FADS2Ex5_20031R |
| FADS2Ex5_20031R | GAG TGG AAG GAA ATG GCT CA | |  |  |  |
| FADS2Ex6/7_27503F | TGA CTT GAC TCC GAG CTT CA | | 1024 | 60°C, 1.5 | FADS2 Ex6.7_27503F |
| FADS2Ex6/7_28526R | CAT CAC TCA GCC TTC AGG AAC | |  |  |  |
| FADS2Ex8_33327F | GGG GTG TGT GCA CAT TGT AG | | 777 | 62°C, 1.5 | FADS2Ex8_33327F |
| FADS2Ex8_34103R | TGC TGA ACC AGT CAC GGT AG | |  |  |  |
| FADS2Ex9/10_33753F | GCC TGA GTT TTG CTG GCT AC | | 878 | 56°C, 1.5 | FADS2Ex9/10_33753F |
| FADS2Ex9/10_34624R | GGG GGT GGG AAA TAC AGT CT | |  |  |  |
| FADS2Ex11_34514F | GGC ATA CAG TGG AGG CAA GT | | 764 | 62°C, 2.0 | FADS2Ex11_34514F |
| FADS2Ex11_35277R | CCT CTC AGC TCT CCA TCA GG | |  |  |  |
| FADS2Ex12a_35258F | CCT GAT GGA GAG CTG AGA GG | | 964 | 58°C, 1.8 | FADS2Ex12a_35258F |
| FADS2Ex12a_36221R | TCC GAC TGC TCC AGA ATC TT | |  |  |  |
| FADS2Ex12b_36124F | AAG AGC TCA GCA GAA GCA ATG | | 870 | 60°C, 1.5 | FADS2Ex12b_36124F |
| FADS2Ex12b_36993R | GTG TAG AGG TGG GGA AGC AA | |  |  |  |

^1^Primer name indicates the gene name and region of the gene amplified by PCR.

^2^Annealing temperature
