# Supplementary material for: Associations between variants of FADS genes and omega-3 and omega-6 milk fatty acids of Canadian Holstein cows
Source: BMC Genet. 2014 Feb 17;15:25. doi: 10.1186/1471-2156-15-25 (PMC3929906; doi:10.1186/1471-2156-15-25)
Supplement: Additional file 2: Table S2 — Primers and probes used in the TaqMan assay for SNP genotyping. [file 1471-2156-15-25-S2.docx]

Table S2: Primers^1^ and probes^2^ used in the TaqMan assay for SNP genotyping

| **Assay name** | **SNP, rs#** | **Forward primer/Reporter 1 sequence** | **Reverse primer/Reporter 2 sequence** |
| --- | --- | --- | --- |
| FADS1-01 | C>T, rs136261927 | CCCCTTAGGAGGCCACTGA/ CAACTCTCCCGCTGTGTC | AGAAGCCACAGACAATGGAACAG/ CAACTCTCCCACTGTGTC |
| FADS1-07 | T>C, rs42187261 | GCCTGGATGATCACTTTCTACGT/ AGCGGCACATAAGTGA | CCCCAGGAAGCCTTTCAGT/ CGGCACGTAAGTGA |
| FADS1-08 | C>T, rs41652284 | CCAGATTGAGCACCAGTGAGTAG/ CCCTGCTACGTGAACC | TGGCTCCAGCTCCTCTGT/ CCTGCTACATGAACC |
| FADS2-05 | C>T, rs211263660 | GCGAGTGGCAGCCCATT/ CCCCTTCATCGTACCTC | CGATTACTGTGTGAGCCAAACG/ CCCTTCATCATACCTC |
| FADS2-14 | C>T, rs211580559 | TCCAGTACCAGATCATCATGACCAT/ TGGACTCACCGCCCAGT | GCCCTCCTCCCCACACT/ TGGACTCACCACCCAGT |
| FADS2-19 | G>A, rs210169303 | GGTCTTTCCTCTCTTGCTGTCT/ TTTCGAGGCATTCACG | GGAGGGTGTCGGAGGGA/ TTCGAGGCGTTCACG |
| FADS2-23 | G>A, rs109772589 | GGAGGGACCAGCTCTGACT/ CCCTCAGGTCCATCAC | CTGATCCCTTGTTGCCATGG/ CCTCAGGCCCATCAC |

^1^Assay mix concentration was 40x, forward and reverse primer concentrations were 36 μM each.

^2^Reporter sequence 1 was labeled with fluorescent VIC dye and reporter sequence 2 with FAM dye. Reporter sequences were at a concentration of 8 μM each.
