# Supplementary material for: Associations between variants of FADS genes and omega-3 and omega-6 milk fatty acids of Canadian Holstein cows
Source: BMC Genet. 2014 Feb 17;15:25. doi: 10.1186/1471-2156-15-25 (PMC3929906; doi:10.1186/1471-2156-15-25)
Supplement: Additional file 3: Table S3 — Identified SNPs within studied regions of FADS1 and FADS2 genes and functional annotation. [file 1471-2156-15-25-S3.docx]

Table S3: Identified SNPs within studied regions of FADS1 and FADS2 genes and functional annotation

| **Gene** | **SNP^1^** | | | | **Functional class** | |
| --- | --- | --- | --- | --- | --- | --- |
|  | Lab name | ss# | rs# |  | Location | Characteristic |
| FADS1 | FADS1-01 | [ss469335027](http://www.ncbi.nlm.nih.gov/SNP/snp_ss.cgi?ss=469335027) | rs136261927 | C/T | Intron 3 |  |
|  | FADS1-02 | [ss469335028](http://www.ncbi.nlm.nih.gov/SNP/snp_ss.cgi?ss=469335028) | rs209573794 | C/A | Intron 6 |  |
|  | FADS1-03 | [ss469335029](http://www.ncbi.nlm.nih.gov/SNP/snp_ss.cgi?ss=469335029) | rs43205274 | C/T | Intron 6 |  |
|  | FADS1-04 | [ss469335030](http://www.ncbi.nlm.nih.gov/SNP/snp_ss.cgi?ss=469335030) | rs208293541 | T/C | Intron 6 |  |
|  | FADS1-05 | [ss469335031](http://www.ncbi.nlm.nih.gov/SNP/snp_ss.cgi?ss=469335031) | rs42187265 | C/T | Intron 6 |  |
|  | FADS1-06 | [ss469335032](http://www.ncbi.nlm.nih.gov/SNP/snp_ss.cgi?ss=469335032) | rs109940390 | A/G | Intron 7 |  |
|  | FADS1-07 | [ss469335033](http://www.ncbi.nlm.nih.gov/SNP/snp_ss.cgi?ss=469335033) | rs42187261 | T/C | Exon 8 | 306 Tyr>Tyr (TA**T**>TA**C**) |
|  | FADS1-08 | [ss469335034](http://www.ncbi.nlm.nih.gov/SNP/snp_ss.cgi?ss=469335034) | rs41652284 | C/T | Intron 10 |  |
|  | FADS1-09 | [ss469335035](http://www.ncbi.nlm.nih.gov/SNP/snp_ss.cgi?ss=469335035) | rs42187260 | C/T | Intron 11 |  |
| FADS2 | FADS2-01 | [ss469335036](http://www.ncbi.nlm.nih.gov/SNP/snp_ss.cgi?ss=469335036) | rs42187018 | C/T | Intron 1 |  |
|  | FADS2-02 | [ss469335037](http://www.ncbi.nlm.nih.gov/SNP/snp_ss.cgi?ss=469335037) | rs134697983 | G/C | Intron 1 |  |
|  | FADS2-03 | [ss469335038](http://www.ncbi.nlm.nih.gov/SNP/snp_ss.cgi?ss=469335038) | rs208449552 | C/T | Intron 2 |  |
|  | FADS2-04 | [ss469335039](http://www.ncbi.nlm.nih.gov/SNP/snp_ss.cgi?ss=469335039) | rs209202414 | A/G | Intron 3 |  |
|  | FADS2-05 | [ss469335040](http://www.ncbi.nlm.nih.gov/SNP/snp_ss.cgi?ss=469335040) | rs211263660 | C/T | Intron 5 | Splice site |
|  | FADS2-06 | [ss469335041](http://www.ncbi.nlm.nih.gov/SNP/snp_ss.cgi?ss=469335041) | rs42187007 | C/T | Intron 5 |  |
|  | FADS2-07 | [ss469335042](http://www.ncbi.nlm.nih.gov/SNP/snp_ss.cgi?ss=469335042) | rs208951880 | G/A | Intron 5 |  |
|  | FADS2-08 | [ss469335043](http://www.ncbi.nlm.nih.gov/SNP/snp_ss.cgi?ss=469335043) | rs110571020 | A/G | Intron 5 |  |
|  | FADS2-09 | [ss469335044](http://www.ncbi.nlm.nih.gov/SNP/snp_ss.cgi?ss=469335044) | rs208102293 | T/C | Intron 5 |  |
|  | FADS2-10 | [ss469335045](http://www.ncbi.nlm.nih.gov/SNP/snp_ss.cgi?ss=469335045) | rs209766856 | G/A | Intron 6 |  |
|  | FADS2-11 | [ss469335046](http://www.ncbi.nlm.nih.gov/SNP/snp_ss.cgi?ss=469335046) | rs109467658 | G/A | Intron 6 |  |
|  | FADS2-12 | [ss469335047](http://www.ncbi.nlm.nih.gov/SNP/snp_ss.cgi?ss=469335047) | rs110795523 | G/A | Intron 6 |  |
|  | FADS2-13 | [ss469335048](http://www.ncbi.nlm.nih.gov/SNP/snp_ss.cgi?ss=469335048) | rs109968079 | A/G | Intron 6 |  |
|  | FADS2-14 | [ss469335049](http://www.ncbi.nlm.nih.gov/SNP/snp_ss.cgi?ss=469335049) | rs211580559 | C/T | Exon 7 | 294 Ala>Val (G**C**G>G**T**G) |
|  | FADS2-15 | [ss469335050](http://www.ncbi.nlm.nih.gov/SNP/snp_ss.cgi?ss=469335050) | rs210475904 | C/T | Intron 7 |  |
|  | FADS2-16 | [ss469335051](http://www.ncbi.nlm.nih.gov/SNP/snp_ss.cgi?ss=469335051) | [rs42186974](http://www.ncbi.nlm.nih.gov/projects/SNP/snp_ref.cgi?rs=42186974) | C/G | Intron 8 |  |
|  | FADS2-17 | [ss469335052](http://www.ncbi.nlm.nih.gov/SNP/snp_ss.cgi?ss=469335052) | rs110328349 | C/T | Intron 9 |  |
|  | FADS2-18 | [ss469335053](http://www.ncbi.nlm.nih.gov/SNP/snp_ss.cgi?ss=469335053) | rs42186973 | G/A | 3'UTR |  |
|  | FADS2-19 | [ss469335054](http://www.ncbi.nlm.nih.gov/SNP/snp_ss.cgi?ss=469335054) | rs210169303 | G/A | 3'UTR | microRNA binding site (bta-miR-744) |
|  | FADS2-20 | [ss469335055](http://www.ncbi.nlm.nih.gov/SNP/snp_ss.cgi?ss=469335055) | rs110020495 | G/A | 3'UTR |  |
|  | FADS2-21 | [ss469335056](http://www.ncbi.nlm.nih.gov/SNP/snp_ss.cgi?ss=469335056) | rs42186972 | C/T | 3'UTR |  |
|  | FADS2-22 | [ss469335057](http://www.ncbi.nlm.nih.gov/SNP/snp_ss.cgi?ss=469335057) | rs210174048 | C/A | 3'UTR |  |
|  | FADS2-23 | [ss469335058](http://www.ncbi.nlm.nih.gov/SNP/snp_ss.cgi?ss=469335058) | rs109772589 | G/A | 3'UTR |  |
|  | FADS2-24 | [ss469335261](http://www.ncbi.nlm.nih.gov/SNP/snp_ss.cgi?ss=469335261) | rs207932003 | G/A | 3'UTR |  |

^1^ss# = submitted SNP number, rs# = reference SNP ID number
