# Supplementary material for: Associations between variants of FADS genes and omega-3 and omega-6 milk fatty acids of Canadian Holstein cows
Source: BMC Genet. 2014 Feb 17;15:25. doi: 10.1186/1471-2156-15-25 (PMC3929906; doi:10.1186/1471-2156-15-25)
Supplement: Additional file 4: Table S4 — Complete marker-trait association results. Significant P-values (P ≤ 0.05) are highlighted yellow. [file 1471-2156-15-25-S4.rtf]

Table S4: Complete marker-trait association results. Significant P-values (P<0.05) are highlighted yellow 

Polyunsaturated Fatty Acids

CLA:9c11t		
SNP	rs#	CC	CT	TT	AA	AG	GG	sem_max	ProbF	FDR-P	
FADS1-01	rs136261927	0.47681	0.47819	0.44711	.	.	.	0.033511	0.6625	0.86359	
FADS1-07	rs42187261	0.47011	0.4787	0.46807	.	.	.	0.014200	0.6505	0.86359	
FADS1-08	rs41652284	0.48132	0.4731	0.47177	.	.	.	0.024530	0.7402	0.86359	
FADS2-05	rs211263660	0.46815	0.4836	0.47033	.	.	.	0.014663	0.3797	0.86359	
FADS2-14	rs211580559	0.46763	0.4793	0.46917	.	.	.	0.013962	0.5746	0.86359	
FADS2-19	rs210169303	0.48233	0.4699	0.43979	.	.	.	0.019459	0.0880	0.61566	
FADS2-23	rs109772589	.	.	.	0.47619	0.47778	0.47926	0.032424	0.9876	0.98760	
CLA:10t12c		
SNP	rs#	CC	CT	TT	AA	AG	GG	sem_max	ProbF	FDR-P	
FADS1-01	rs136261927	0.02343	0.02345	0.02119	.	.	.	.00182912	0.4702	0.66317	
FADS1-07	rs42187261	0.02391	0.02331	0.02330	.	.	.	.00081976	0.7624	0.76243	
FADS1-08	rs41652284	0.02375	0.02402	0.02278	.	.	.	.00128925	0.6262	0.73061	
FADS2-05	rs211263660	0.02267	0.02398	0.02304	.	.	.	.00083132	0.1225	0.32821	
FADS2-14	rs211580559	0.02273	0.02397	0.02292	.	.	.	.00079203	0.1407	0.32821	
FADS2-19	rs210169303	0.02392	0.02302	0.02096	.	.	.	.00110814	0.0271	0.18950	
FADS2-23	rs109772589	.	.	.	0.02339	0.02333	0.02556	.00182887	0.4737	0.66317	
C18:2n6cc		
SNP	rs#	CC	CT	TT	AA	AG	GG	sem_max	ProbF	FDR-P	
FADS1-01	rs136261927	1.86474	1.83015	1.87308	.	.	.	0.085249	0.4848	0.99228	
FADS1-07	rs42187261	1.90414	1.84815	1.81839	.	.	.	0.035586	0.1047	0.40011	
FADS1-08	rs41652284	1.88176	1.82996	1.80076	.	.	.	0.061784	0.1143	0.40011	
FADS2-05	rs211263660	1.84948	1.83502	1.86626	.	.	.	0.036953	0.7005	0.99228	
FADS2-14	rs211580559	1.85035	1.84676	1.84939	.	.	.	0.035289	0.9923	0.99228	
FADS2-19	rs210169303	1.84063	1.84527	1.86306	.	.	.	0.049590	0.9061	0.99228	
FADS2-23	rs109772589	.	.	.	1.84906	1.83631	1.88419	0.082433	0.8044	0.99228	
C18:2n6tt		
SNP	rs#	CC	CT	TT	AA	AG	GG	sem_max	ProbF	FDR-P	
FADS1-01	rs136261927	0.16273	0.15931	0.15749	.	.	.	0.014098	0.7452	0.99456	
FADS1-07	rs42187261	0.15845	0.15732	0.17027	.	.	.	0.005908	0.0339	0.17640	
FADS1-08	rs41652284	0.16070	0.16064	0.15963	.	.	.	0.010289	0.9946	0.99456	
FADS2-05	rs211263660	0.16370	0.15891	0.16074	.	.	.	0.006181	0.6251	0.99456	
FADS2-14	rs211580559	0.16155	0.16003	0.16169	.	.	.	0.005793	0.9375	0.99456	
FADS2-19	rs210169303	0.16492	0.15781	0.14659	.	.	.	0.008011	0.0504	0.17640	
FADS2-23	rs109772589	.	.	.	0.15807	0.16383	0.17466	0.013529	0.2720	0.63470	
C18:3ntcc		
SNP	rs#	CC	CT	TT	AA	AG	GG	sem_max	ProbF	FDR-P	
FADS1-01	rs136261927	0.10870	0.10725	0.10197	.	.	.	.00688037	0.5451	0.76314	
FADS1-07	rs42187261	0.10767	0.10729	0.10729	.	.	.	.00289809	0.9911	0.99110	
FADS1-08	rs41652284	0.10754	0.10709	0.10351	.	.	.	.00493980	0.7154	0.83462	
FADS2-05	rs211263660	0.10497	0.10960	0.10660	.	.	.	.00295369	0.1407	0.49260	
FADS2-14	rs211580559	0.10516	0.10856	0.10599	.	.	.	.00285129	0.3415	0.76314	
FADS2-19	rs210169303	0.10803	0.10819	0.09983	.	.	.	.00397681	0.1167	0.49260	
FADS2-23	rs109772589	.	.	.	0.10663	0.10922	0.10944	.00662770	0.5222	0.76314	
C18:3n3		
SNP	rs#	CC	CT	TT	AA	AG	GG	sem_max	ProbF	FDR-P	
FADS1-01	rs136261927	0.42456	0.40973	0.42246	.	.	.	0.023918	0.1957	0.45653	
FADS1-07	rs42187261	0.43032	0.42074	0.40960	.	.	.	0.010076	0.1725	0.45653	
FADS1-08	rs41652284	0.42650	0.41933	0.39658	.	.	.	0.017114	0.1821	0.45653	
FADS2-05	rs211263660	0.41497	0.41875	0.42167	.	.	.	0.010349	0.8154	0.83544	
FADS2-14	rs211580559	0.41586	0.42067	0.41714	.	.	.	0.009800	0.8354	0.83544	
FADS2-19	rs210169303	0.42115	0.41696	0.40136	.	.	.	0.014072	0.3853	0.53947	
FADS2-23	rs109772589	.	.	.	0.41523	0.42204	0.44673	0.023085	0.3176	0.53947	
C20:3n6		
SNP	rs#	CC	CT	TT	AA	AG	GG	sem_max	ProbF	FDR-P	
FADS1-01	rs136261927	0.09548	0.08734	0.07549	.	.	.	.00720931	0.0003	0.00200	
FADS1-07	rs42187261	0.08992	0.08900	0.09618	.	.	.	.00310548	0.0241	0.05616	
FADS1-08	rs41652284	0.09474	0.08899	0.08942	.	.	.	.00533731	0.0546	0.09548	
FADS2-05	rs211263660	0.09381	0.09022	0.09470	.	.	.	.00316167	0.2388	0.28667	
FADS2-14	rs211580559	0.09359	0.08983	0.09324	.	.	.	.00303155	0.2867	0.28667	
FADS2-19	rs210169303	0.09262	0.09057	0.08556	.	.	.	.00433153	0.2594	0.28667	
FADS2-23	rs109772589	.	.	.	0.08915	0.09648	0.1084 3	.00697630	0.0008	0.00286	
C20:4n6		
SNP	rs#	CC	CT	TT	AA	AG	GG	sem_max	ProbF	FDR-P	
FADS1-01	rs136261927	0.11849	0.11621	0.11873	.	.	.	.00702884	0.6367	0.63666	
FADS1-07	rs42187261	0.11411	0.11586	0.12186	.	.	.	.00286888	0.0211	0.07391	
FADS1-08	rs41652284	0.11970	0.11468	0.12140	.	.	.	.00505267	0.0708	0.16523	
FADS2-05	rs211263660	0.12061	0.11609	0.11622	.	.	.	.00300603	0.1445	0.25288	
FADS2-14	rs211580559	0.11996	0.11622	0.11582	.	.	.	.00287782	0.2437	0.34121	
FADS2-19	rs210169303	0.11744	0.11642	0.12086	.	.	.	.00404873	0.5834	0.63666	
FADS2-23	rs109772589	.	.	.	0.11495	0.12264	0.12648	.00653771	0.0019	0.01298	
C20:5n3		
SNP	rs#	CC	CT	TT	AA	AG	GG	sem_max	ProbF	FDR-P	
FADS1-01	rs136261927	0.03473	0.03464	0.03586	.	.	.	.00355770	0.9463	0.94629	
FADS1-07	rs42187261	0.03578	0.03312	0.03726	.	.	.	.00147865	0.0041	0.02893	
FADS1-08	rs41652284	0.03487	0.03419	0.03985	.	.	.	.00256709	0.0951	0.33286	
FADS2-05	rs211263660	0.03425	0.03524	0.03333	.	.	.	.00156961	0.4592	0.80357	
FADS2-14	rs211580559	0.03453	0.03487	0.03334	.	.	.	.00149671	0.6144	0.86023	
FADS2-19	rs210169303	0.03460	0.03478	0.03399	.	.	.	.00216321	0.9412	0.94629	
FADS2-23	rs109772589	.	.	.	0.03416	0.03509	0.03902	.00338359	0.3008	0.70191	
C22:5n3		
SNP	rs#	CC	CT	TT	AA	AG	GG	sem_max	ProbF	FDR-P	
FADS1-01	rs136261927	0.08068	0.09122	0.05492	.	.	.	0.038678	0.5528	0.90429	
FADS1-07	rs42187261	0.09156	0.07771	0.09491	.	.	.	0.015884	0.4051	0.90429	
FADS1-08	rs41652284	0.08206	0.08134	0.10674	.	.	.	0.027401	0.6484	0.90429	
FADS2-05	rs211263660	0.08100	0.09002	0.07171	.	.	.	0.016670	0.5373	0.90429	
FADS2-14	rs211580559	0.08253	0.08311	0.08997	.	.	.	0.016145	0.9036	0.90429	
FADS2-19	rs210169303	0.08077	0.09320	0.07578	.	.	.	0.021761	0.5595	0.90429	
FADS2-23	rs109772589	.	.	.	0.08352	0.08784	0.07432	0.036648	0.9043	0.90429	
Total PUFA		
SNP	rs#	CC	CT	TT	AA	AG	GG	sem_max	ProbF	FDR-P	
FADS1-01	rs136261927	3.29295	3.24204	3.28458	.	.	.	0.15035	0.6137	0.85913	
FADS1-07	rs42187261	3.24648	3.27987	3.25032	.	.	.	0.06315	0.8098	0.92062	
FADS1-08	rs41652284	3.31723	3.24154	3.21933	.	.	.	0.10817	0.2528	0.80629	
FADS2-05	rs211263660	3.28409	3.27020	3.19411	.	.	.	0.06501	0.4224	0.80629	
FADS2-14	rs211580559	3.28151	3.27842	3.18003	.	.	.	0.06135	0.2524	0.80629	
FADS2-19	rs210169303	3.25188	3.27218	3.25181	.	.	.	0.08793	0.9206	0.92062	
FADS2-23	rs109772589	.	.	.	3.25237	3.28228	3.42056	0.14427	0.4607	0.80629	


Monounsaturated Fatty Acids

C14:1		
SNP	rs#	CC	CT	TT	AA	AG	GG	sem_max	ProbF	FDR-P	
FADS1-01	rs136261927	1.03824	1.02307	1.02875	.	.	.	0.10261	0.9101	0.91014	
FADS1-07	rs42187261	0.98113	1.02059	1.05552	.	.	.	0.04258	0.2964	0.47389	
FADS1-08	rs41652284	1.03833	0.99116	1.14891	.	.	.	0.07261	0.0689	0.47389	
FADS2-05	rs211263660	1.06118	1.02057	0.99844	.	.	.	0.04402	0.3385	0.47389	
FADS2-14	rs211580559	1.05507	1.01658	0.98675	.	.	.	0.04190	0.2956	0.47389	
FADS2-19	rs210169303	1.01690	1.03981	1.00840	.	.	.	0.05910	0.7667	0.89445	
FADS2-23	rs109772589	.	.	.	1.01383	1.06830	0.95848	0.09861	0.2164	0.47389	
C14:1t		
SNP	rs#	CC	CT	TT	AA	AG	GG	sem_max	ProbF	FDR-P	
FADS1-01	rs136261927	0.25470	0.24727	0.23254	.	.	.	0.020316	0.3512	0.35122	
FADS1-07	rs42187261	0.26056	0.24735	0.25159	.	.	.	0.008504	0.3276	0.35122	
FADS1-08	rs41652284	0.25626	0.24757	0.24182	.	.	.	0.014628	0.3215	0.35122	
FADS2-05	rs211263660	0.24449	0.25289	0.26317	.	.	.	0.008823	0.1370	0.35122	
FADS2-14	rs211580559	0.24481	0.24984	0.26098	.	.	.	0.008484	0.2194	0.35122	
FADS2-19	rs210169303	0.25324	0.24978	0.23483	.	.	.	0.011935	0.3220	0.35122	
FADS2-23	rs109772589	.	.	.	0.24669	0.25902	0.21346	0.019480	0.0291	0.20354	
C16:1		
SNP	rs#	CC	CT	TT	AA	AG	GG	sem_max	ProbF	FDR-P	
FADS1-01	rs136261927	1.84185	1.82880	1.82653	.	.	.	0.12428	0.9502	0.98414	
FADS1-07	rs42187261	1.84478	1.83695	1.84352	.	.	.	0.05233	0.9841	0.98414	
FADS1-08	rs41652284	1.85151	1.80697	1.80821	.	.	.	0.09049	0.5358	0.98414	
FADS2-05	rs211263660	1.86202	1.81289	1.83775	.	.	.	0.05365	0.5196	0.98414	
FADS2-14	rs211580559	1.86574	1.82889	1.82081	.	.	.	0.05129	0.6241	0.98414	
FADS2-19	rs210169303	1.84213	1.84123	1.81899	.	.	.	0.07239	0.9516	0.98414	
FADS2-23	rs109772589	.	.	.	1.84721	1.85660	1.83615	0.11936	0.9678	0.98414	
C16:1t		
SNP	rs#	CC	CT	TT	AA	AG	GG	sem_max	ProbF	FDR-P	
FADS1-01	rs136261927	0.33255	0.32679	0.32188	.	.	.	0.014596	0.4283	0.59960	
FADS1-07	rs42187261	0.33486	0.33124	0.32366	.	.	.	0.006114	0.2055	0.59960	
FADS1-08	rs41652284	0.33495	0.32806	0.32303	.	.	.	0.010469	0.2391	0.59960	
FADS2-05	rs211263660	0.32508	0.33234	0.32898	.	.	.	0.006326	0.3590	0.59960	
FADS2-14	rs211580559	0.32689	0.33070	0.32936	.	.	.	0.006015	0.7541	0.75409	
FADS2-19	rs210169303	0.33129	0.32719	0.32880	.	.	.	0.008500	0.7082	0.75409	
FADS2-23	rs109772589	.	.	.	0.32782	0.33432	0.32868	0.013891	0.4129	0.59960	
C18:1n9c		
SNP	rs#	CC	CT	TT	AA	AG	GG	sem_max	ProbF	FDR-P	
FADS1-01	rs136261927	21.0357	20.8392	19.8338	.	.	.	1.20482	0.5695	0.56952	
FADS1-07	rs42187261	21.4189	21.3742	20.0895	.	.	.	0.51964	0.0106	0.07416	
FADS1-08	rs41652284	20.9468	21.2525	19.1479	.	.	.	0.89421	0.0687	0.24043	
FADS2-05	rs211263660	20.4158	21.0773	21.2118	.	.	.	0.51838	0.2008	0.46864	
FADS2-14	rs211580559	20.5963	21.2005	20.9972	.	.	.	0.51680	0.3835	0.56952	
FADS2-19	rs210169303	21.1951	20.9254	20.4395	.	.	.	0.73436	0.5577	0.56952	
FADS2-23	rs109772589	.	.	.	21.0659	20.9915	22.3703	1.19937	0.5227	0.56952	
C18:1n9t		
SNP	rs#	CC	CT	TT	AA	AG	GG	sem_max	ProbF	FDR-P	
FADS1-01	rs136261927	0.30571	0.32793	0.31916	.	.	.	0.070113	0.6495	0.64950	
FADS1-07	rs42187261	0.28748	0.32804	0.29971	.	.	.	0.029651	0.3227	0.56466	
FADS1-08	rs41652284	0.29625	0.33182	0.28405	.	.	.	0.050908	0.2664	0.56466	
FADS2-05	rs211263660	0.30041	0.32640	0.27732	.	.	.	0.030398	0.2546	0.56466	
FADS2-14	rs211580559	0.30295	0.32448	0.27568	.	.	.	0.028733	0.2503	0.56466	
FADS2-19	rs210169303	0.31910	0.30369	0.28199	.	.	.	0.042003	0.6271	0.64950	
FADS2-23	rs109772589	.	.	.	0.30084	0.33146	0.31160	0.067959	0.4427	0.61973	
C18:1n11t		
SNP	rs#	CC	CT	TT	AA	AG	GG	sem_max	ProbF	FDR-P	
FADS1-01	rs136261927	1.42205	1.39877	1.44982	.	.	.	0.16680	0.9008	0.97959	
FADS1-07	rs42187261	1.40912	1.41902	1.40772	.	.	.	0.07032	0.9796	0.97959	
FADS1-08	rs41652284	1.41324	1.41542	1.36871	.	.	.	0.11804	0.9251	0.97959	
FADS2-05	rs211263660	1.41189	1.43085	1.34433	.	.	.	0.07227	0.5162	0.97959	
FADS2-14	rs211580559	1.39103	1.43548	1.32543	.	.	.	0.06842	0.2955	0.97959	
FADS2-19	rs210169303	1.44030	1.34513	1.42923	.	.	.	0.09887	0.2486	0.97959	
FADS2-23	rs109772589	.	.	.	1.40913	1.39555	1.50878	0.16159	0.7829	0.97959	
C18:1 total		
SNP	rs#	CC	CT	TT	AA	AG	GG	sem_max	ProbF	FDR-P	
FADS1-01	rs136261927	24.1021	23.8568	22.7231	.	.	.	1.26587	0.5012	0.50645	
FADS1-07	rs42187261	24.4587	24.4385	23.1060	.	.	.	0.54372	0.0120	0.08395	
FADS1-08	rs41652284	24.0026	24.2828	22.0761	.	.	.	0.93828	0.0696	0.24374	
FADS2-05	rs211263660	23.4541	24.1358	24.1583	.	.	.	0.54625	0.2504	0.50645	
FADS2-14	rs211580559	23.6265	24.2655	23.9298	.	.	.	0.54146	0.3758	0.50645	
FADS2-19	rs210169303	24.2649	23.9019	23.4569	.	.	.	0.76768	0.4980	0.50645	
FADS2-23	rs109772589	.	.	.	24.0741	24.0842	25.5245	1.25533	0.5065	0.50645	
Total MUFA		
SNP	rs#	CC	CT	TT	AA	AG	GG	sem_max	ProbF	FDR-P	
FADS1-01	rs136261927	30.4159	29.8701	28.6180	.	.	.	1.44145	0.2870	0.66957	
FADS1-07	rs42187261	30.5981	30.6690	29.2074	.	.	.	0.61968	0.0187	0.13124	
FADS1-08	rs41652284	30.3629	30.2526	28.2513	.	.	.	1.06560	0.1393	0.48760	
FADS2-05	rs211263660	29.7757	30.2208	30.3749	.	.	.	0.62457	0.5739	0.74691	
FADS2-14	rs211580559	29.9629	30.3553	30.1302	.	.	.	0.61223	0.7469	0.74691	
FADS2-19	rs210169303	30.3809	30.2660	29.2662	.	.	.	0.86995	0.4600	0.74691	
FADS2-23	rs109772589	.	.	.	30.2777	30.2829	31.4547	1.42643	0.7062	0.74691	


Saturated Fatty acids

C4:0		
SNP	rs#	CC	CT	TT	AA	AG	GG	sem_max	ProbF	FDR-P	
FADS1-01	rs136261927	0.79761	0.81964	0.84960	.	.	.	0.042630	0.1837	0.38771	
FADS1-07	rs42187261	0.81150	0.80667	0.81604	.	.	.	0.017746	0.8272	0.96382	
FADS1-08	rs41652284	0.79826	0.82547	0.79207	.	.	.	0.030633	0.1267	0.38771	
FADS2-05	rs211263660	0.81063	0.80970	0.80079	.	.	.	0.018304	0.8724	0.96382	
FADS2-14	rs211580559	0.81130	0.80742	0.81020	.	.	.	0.017441	0.9638	0.96382	
FADS2-19	rs210169303	0.81390	0.79134	0.83448	.	.	.	0.024449	0.1385	0.38771	
FADS2-23	rs109772589	.	.	.	0.81223	0.79283	0.84859	0.040684	0.2215	0.38771	
C6:0		
SNP	rs#	CC	CT	TT	AA	AG	GG	sem_max	ProbF	FDR-P	
FADS1-01	rs136261927	1.00352	1.02077	1.03451	.	.	.	0.046897	0.4840	0.82773	
FADS1-07	rs42187261	1.00399	1.00127	1.04101	.	.	.	0.019748	0.0563	0.39376	
FADS1-08	rs41652284	1.00624	1.02288	1.01482	.	.	.	0.033940	0.5588	0.82773	
FADS2-05	rs211263660	1.01965	1.01312	1.00154	.	.	.	0.020271	0.7095	0.82773	
FADS2-14	rs211580559	1.01721	1.00988	1.01781	.	.	.	0.019487	0.8772	0.87722	
FADS2-19	rs210169303	1.01174	1.00194	1.03275	.	.	.	0.027102	0.5407	0.82773	
FADS2-23	rs109772589	.	.	.	1.01367	1.00005	1.01564	0.044935	0.6821	0.82773	
C8:0		
SNP	rs#	CC	CT	TT	AA	AG	GG	sem_max	ProbF	FDR-P	
FADS1-01	rs136261927	0.88854	0.89899	0.89285	.	.	.	0.045758	0.8006	0.92493	
FADS1-07	rs42187261	0.87630	0.88299	0.92109	.	.	.	0.019388	0.0431	0.30146	
FADS1-08	rs41652284	0.89410	0.89614	0.90714	.	.	.	0.033460	0.9249	0.92493	
FADS2-05	rs211263660	0.90010	0.89304	0.89160	.	.	.	0.019783	0.8814	0.92493	
FADS2-14	rs211580559	0.89633	0.89017	0.90510	.	.	.	0.019191	0.7502	0.92493	
FADS2-19	rs210169303	0.89172	0.88689	0.89953	.	.	.	0.026896	0.8931	0.92493	
FADS2-23	rs109772589	.	.	.	0.89387	0.88313	0.88634	0.044387	0.7883	0.92493	
C10:0		
SNP	rs#	CC	CT	TT	AA	AG	GG	sem_max	ProbF	FDR-P	
FADS1-01	rs136261927	2.61610	2.63244	2.56406	.	.	.	0.16298	0.9011	0.98878	
FADS1-07	rs42187261	2.54100	2.59162	2.70626	.	.	.	0.06887	0.0631	0.44161	
FADS1-08	rs41652284	2.64188	2.60680	2.72066	.	.	.	0.11967	0.5922	0.98878	
FADS2-05	rs211263660	2.63549	2.61544	2.66463	.	.	.	0.07049	0.7912	0.98878	
FADS2-14	rs211580559	2.62217	2.60865	2.69411	.	.	.	0.06858	0.4809	0.98878	
FADS2-19	rs210169303	2.61621	2.61001	2.60393	.	.	.	0.09603	0.9888	0.98878	
FADS2-23	rs109772589	.	.	.	2.62315	2.59320	2.54443	0.15917	0.7921	0.98878	
C11:0		
SNP	rs#	CC	CT	TT	AA	AG	GG	sem_max	ProbF	FDR-P	
FADS1-01	rs136261927	0.22435	0.22954	0.23409	.	.	.	0.017874	0.6302	0.83051	
FADS1-07	rs42187261	0.21700	0.22136	0.23760	.	.	.	0.007474	0.0166	0.11645	
FADS1-08	rs41652284	0.22525	0.22468	0.24915	.	.	.	0.012709	0.1537	0.53781	
FADS2-05	rs211263660	0.23229	0.22525	0.22014	.	.	.	0.007667	0.2892	0.67487	
FADS2-14	rs211580559	0.23015	0.22360	0.22158	.	.	.	0.007399	0.4652	0.81408	
FADS2-19	rs210169303	0.22431	0.22632	0.22503	.	.	.	0.010547	0.9481	0.94806	
FADS2-23	rs109772589	.	.	.	0.22412	0.22836	0.21821	0.017311	0.7119	0.83051	
C12:0		
SNP	rs#	CC	CT	TT	AA	AG	GG	sem_max	ProbF	FDR-P	
FADS1-01	rs136261927	3.42308	3.42544	3.32376	.	.	.	0.22591	0.9060	0.96214	
FADS1-07	rs42187261	3.28572	3.38208	3.52847	.	.	.	0.09471	0.0571	0.40003	
FADS1-08	rs41652284	3.46043	3.37325	3.63563	.	.	.	0.16478	0.2170	0.75934	
FADS2-05	rs211263660	3.44791	3.40481	3.48953	.	.	.	0.09784	0.6789	0.96214	
FADS2-14	rs211580559	3.42663	3.39468	3.50983	.	.	.	0.09498	0.5085	0.96214	
FADS2-19	rs210169303	3.40986	3.41415	3.37533	.	.	.	0.13310	0.9621	0.96214	
FADS2-23	rs109772589	.	.	.	3.41381	3.39966	3.29734	0.22024	0.8635	0.96214	
C13:0		
SNP	rs#	CC	CT	TT	AA	AG	GG	sem_max	ProbF	FDR-P	
FADS1-01	rs136261927	0.44764	0.41986	0.41923	.	.	.	0.041122	0.1290	0.34962	
FADS1-07	rs42187261	0.42325	0.43380	0.43521	.	.	.	0.016913	0.8005	0.97974	
FADS1-08	rs41652284	0.44561	0.41463	0.44326	.	.	.	0.029470	0.0659	0.34962	
FADS2-05	rs211263660	0.43539	0.43509	0.43495	.	.	.	0.017811	0.9996	0.99964	
FADS2-14	rs211580559	0.42878	0.43664	0.43682	.	.	.	0.016851	0.8398	0.97974	
FADS2-19	rs210169303	0.42257	0.45027	0.43333	.	.	.	0.024296	0.1498	0.34962	
FADS2-23	rs109772589	.	.	.	0.43167	0.44042	0.42465	0.039616	0.7933	0.97974	
C14:0		
SNP	rs#	CC	CT	TT	AA	AG	GG	sem_max	ProbF	FDR-P	
FADS1-01	rs136261927	12.1315	12.2061	11.8411	.	.	.	0.53712	0.7771	0.86780	
FADS1-07	rs42187261	11.9679	12.0474	12.2887	.	.	.	0.22840	0.3597	0.86780	
FADS1-08	rs41652284	12.1856	12.0309	12.4763	.	.	.	0.38939	0.4437	0.86780	
FADS2-05	rs211263660	12.1533	12.1389	12.2649	.	.	.	0.23154	0.8678	0.86780	
FADS2-14	rs211580559	12.1064	12.1007	12.2789	.	.	.	0.22571	0.7284	0.86780	
FADS2-19	rs210169303	12.1381	12.1083	11.8634	.	.	.	0.31960	0.7049	0.86780	
FADS2-23	rs109772589	.	.	.	12.1089	12.1199	11.7907	0.52719	0.8240	0.86780	
C15:0		
SNP	rs#	CC	CT	TT	AA	AG	GG	sem_max	ProbF	FDR-P	
FADS1-01	rs136261927	1.15725	1.11740	1.24088	.	.	.	0.079722	0.1688	0.29545	
FADS1-07	rs42187261	1.13629	1.11694	1.17802	.	.	.	0.032599	0.0961	0.29545	
FADS1-08	rs41652284	1.14695	1.09782	1.26225	.	.	.	0.057692	0.0103	0.07182	
FADS2-05	rs211263660	1.16661	1.12602	1.15608	.	.	.	0.034579	0.3205	0.32049	
FADS2-14	rs211580559	1.15795	1.12054	1.16162	.	.	.	0.032960	0.3000	0.32049	
FADS2-19	rs210169303	1.12107	1.15570	1.18159	.	.	.	0.046436	0.2691	0.32049	
FADS2-23	rs109772589	.	.	.	1.13153	1.16292	1.01830	0.076855	0.1316	0.29545	
C16:0		
SNP	rs#	CC	CT	TT	AA	AG	GG	sem_max	ProbF	FDR-P	
FADS1-01	rs136261927	33.1904	33.7258	35.0421	.	.	.	1.11043	0.1180	0.16526	
FADS1-07	rs42187261	33.1073	33.1624	34.0076	.	.	.	0.46040	0.0770	0.15102	
FADS1-08	rs41652284	33.2013	33.3168	34.9604	.	.	.	0.79429	0.0863	0.15102	
FADS2-05	rs211263660	34.0191	33.2651	32.9159	.	.	.	0.47552	0.0505	0.15102	
FADS2-14	rs211580559	33.8818	33.2416	33.0664	.	.	.	0.45625	0.1547	0.18051	
FADS2-19	rs210169303	33.0489	33.7914	34.1404	.	.	.	0.64328	0.0678	0.15102	
FADS2-23	rs109772589	.	.	.	33.4910	33.3499	31.5612	1.05334	0.1846	0.18456	
C17:0		
SNP	rs#	CC	CT	TT	AA	AG	GG	sem_max	ProbF	FDR-P	
FADS1-01	rs136261927	0.66812	0.65914	0.68801	.	.	.	0.022149	0.2955	0.69497	
FADS1-07	rs42187261	0.65666	0.66857	0.66141	.	.	.	0.009179	0.3971	0.69497	
FADS1-08	rs41652284	0.66893	0.65818	0.69807	.	.	.	0.015761	0.0314	0.21986	
FADS2-05	rs211263660	0.66300	0.66549	0.66607	.	.	.	0.009600	0.9344	0.96337	
FADS2-14	rs211580559	0.66298	0.66506	0.66457	.	.	.	0.009085	0.9634	0.96337	
FADS2-19	rs210169303	0.66179	0.66844	0.67053	.	.	.	0.012852	0.6043	0.84598	
FADS2-23	rs109772589	.	.	.	0.66209	0.67331	0.67331	0.021172	0.3079	0.69497	
C18:0		
SNP	rs#	CC	CT	TT	AA	AG	GG	sem_max	ProbF	FDR-P	
FADS1-01	rs136261927	10.0619	9.8857	10.0942	.	.	.	0.66592	0.7363	0.78411	
FADS1-07	rs42187261	10.2363	10.0676	9.8731	.	.	.	0.27085	0.4746	0.78411	
FADS1-08	rs41652284	9.9916	10.1796	9.5450	.	.	.	0.45970	0.3398	0.78411	
FADS2-05	rs211263660	9.8622	10.0926	10.0927	.	.	.	0.28459	0.5632	0.78411	
FADS2-14	rs211580559	9.9165	10.0470	10.0995	.	.	.	0.27167	0.7841	0.78411	
FADS2-19	rs210169303	10.1312	9.8129	10.2947	.	.	.	0.37503	0.2578	0.78411	
FADS2-23	rs109772589	.	.	.	9.94400	9.97523	10.9227	0.61794	0.2833	0.78411	
C20:0		
SNP	rs#	CC	CT	TT	AA	AG	GG	sem_max	ProbF	FDR-P	
FADS1-01	rs136261927	0.13202	0.13184	0.12928	.	.	.	.007847	0.9411	0.94108	
FADS1-07	rs42187261	0.13225	0.13117	0.13413	.	.	.	.003170	0.5603	0.94108	
FADS1-08	rs41652284	0.13115	0.13461	0.12842	.	.	.	.005522	0.2908	0.94108	
FADS2-05	rs211263660	0.13081	0.13338	0.12998	.	.	.	.003330	0.4929	0.94108	
FADS2-14	rs211580559	0.13148	0.13226	0.13078	.	.	.	.003220	0.8986	0.94108	
FADS2-19	rs210169303	0.13294	0.13077	0.13156	.	.	.	.004429	0.6993	0.94108	
FADS2-23	rs109772589	.	.	.	0.13041	0.13321	0.14240	.007246	0.1748	0.94108	
C22:0		
SNP	rs#	CC	CT	TT	AA	AG	GG	sem_max	ProbF	FDR-P	
FADS1-01	rs136261927	0.05507	0.05377	0.05534	.	.	.	.0034143	0.5323	0.86573	
FADS1-07	rs42187261	0.05566	0.05352	0.05591	.	.	.	.0014268	0.1041	0.72883	
FADS1-08	rs41652284	0.05461	0.05458	0.05449	.	.	.	.0024976	0.9987	0.99865	
FADS2-05	rs211263660	0.05346	0.05540	0.05452	.	.	.	.0014665	0.2582	0.76587	
FADS2-14	rs211580559	0.05378	0.05460	0.05476	.	.	.	.0014182	0.7421	0.86573	
FADS2-19	rs210169303	0.05451	0.05469	0.05277	.	.	.	.0020050	0.6595	0.86573	
FADS2-23	rs109772589	.	.	.	0.05399	0.05495	0.05836	.0032519	0.3282	0.76587	
C23:0		
SNP	rs#	CC	CT	TT	AA	AG	GG	sem_max	ProbF	FDR-P	
FADS1-01	rs136261927	0.02467	0.02449	0.02642	.	.	.	.00327594	0.8479	0.98923	
FADS1-07	rs42187261	0.02416	0.02343	0.02765	.	.	.	.00134184	0.0015	0.01062	
FADS1-08	rs41652284	0.02459	0.02522	0.02628	.	.	.	.00238907	0.7096	0.98923	
FADS2-05	rs211263660	0.02552	0.02451	0.02401	.	.	.	.00140449	0.5461	0.95565	
FADS2-14	rs211580559	0.02585	0.02431	0.02412	.	.	.	.00132640	0.3430	0.80041	
FADS2-19	rs210169303	0.02455	0.02471	0.02452	.	.	.	.00196123	0.9896	0.98959	
FADS2-23	rs109772589	.	.	.	0.02416	0.02570	0.02684	.00309932	0.2993	0.80041	
C24:0		
SNP	rs#	CC	CT	TT	AA	AG	GG	sem_max	ProbF	FDR-P	
FADS1-01	rs136261927	0.04463	0.04522	0.04055	.	.	.	0.012198	0.9316	0.93159	
FADS1-07	rs42187261	0.04025	0.04375	0.04993	.	.	.	0.005232	0.2130	0.49938	
FADS1-08	rs41652284	0.04357	0.04532	0.03671	.	.	.	0.008468	0.5963	0.69568	
FADS2-05	rs211263660	0.04585	0.04603	0.03775	.	.	.	0.005285	0.2854	0.49938	
FADS2-14	rs211580559	0.04589	0.04570	0.03881	.	.	.	0.005235	0.3956	0.55386	
FADS2-19	rs210169303	0.04740	0.04358	0.03602	.	.	.	0.007416	0.2837	0.49938	
FADS2-23	rs109772589	.	.	.	0.04221	0.05059	0.04306	0.011455	0.1195	0.49938	
Total SFA		
SNP	rs#	CC	CT	TT	AA	AG	GG	sem_max	ProbF	FDR-P	
FADS1-01	rs136261927	66.2912	66.8879	68.0974	.	.	.	1.48564	0.2776	0.64769	
FADS1-07	rs42187261	66.1554	66.0511	67.5423	.	.	.	0.63822	0.0209	0.14651	
FADS1-08	rs41652284	66.3199	66.5059	68.5293	.	.	.	1.09795	0.1326	0.46394	
FADS2-05	rs211263660	66.9402	66.5090	66.4310	.	.	.	0.64445	0.6465	0.74113	
FADS2-14	rs211580559	66.7556	66.3663	66.6898	.	.	.	0.63034	0.7411	0.74113	
FADS2-19	rs210169303	66.3672	66.4619	67.4819	.	.	.	0.89609	0.4794	0.74113	
FADS2-23	rs109772589	.	.	.	66.4699	66.4348	65.1248	1.46881	0.6548	0.74113	
