# Supplementary material for: Associations between variants of FADS genes and omega-3 and omega-6 milk fatty acids of Canadian Holstein cows
Source: BMC Genet. 2014 Feb 17;15:25. doi: 10.1186/1471-2156-15-25 (PMC3929906; doi:10.1186/1471-2156-15-25)
Supplement: Additional file 5: Table S5 — Complete results on allele substitution effect. The reference genotype is either CC or GG. Significant p-values (P ≤ 0.05) are highlighted yellow. [file 1471-2156-15-25-S5.rtf]

Table S5: Complete results on allele substitution effect. The reference genotype is either CC or GG. Significant p-values (P≤0.05) are highlighted yellow 


SNP	rs#	1Effect	S.E.	P-Value	FDR P-value		Effect	S.E.	P-Value	FDR P-value	
	Polyunsaturated Fatty Acids		
CLA:9c11t			CLA:10t12c		
FADS1-01	rs136261927	-0.0035	0.0099	0.7255	0.87439		-0.0003	0.0006	0.5425	0.70723	
FADS1-07	rs42187261	-0.0024	0.0079	0.7621	0.87439		-0.0003	0.0004	0.5719	0.70723	
FADS1-08	rs41652284	-0.0067	0.0090	0.4583	0.87439		-0.0001	0.0005	0.8684	0.86844	
FADS2-05	rs211263660	0.0039	0.0076	0.6131	0.87439		0.0004	0.0004	0.3255	0.70723	
FADS2-14	rs211580559	0.0024	0.0075	0.7445	0.87439		0.0003	0.0004	0.4898	0.70723	
FADS2-19	rs210169303	-0.0176	0.0083	0.0355	0.24834		-0.0012	0.0005	0.0100	0.06995	
FADS2-23	rs109772589	-0.0016	0.0099	0.8744	0.87439		-0.0003	0.0006	0.6062	0.70723	
C18:2n6CC			C18:2n6TT		
FADS1-01	rs136261927	-0.0230	0.0252	0.3623	0.84530		-0.0032	0.0042	0.4465	0.71307	
FADS1-07	rs42187261	-0.0410	0.0197	0.0383	0.13716		0.0069	0.0033	0.0360	0.12596	
FADS1-08	rs41652284	-0.0468	0.0226	0.0392	0.13716		-0.0003	0.0038	0.9430	0.95433	
FADS2-05	rs211263660	0.0040	0.0192	0.8370	0.95942		-0.0021	0.0032	0.5093	0.71307	
FADS2-14	rs211580559	-0.0010	0.0188	0.9594	0.95942		-0.0002	0.0031	0.9543	0.95433	
FADS2-19	rs210169303	0.0085	0.0212	0.6894	0.95942		-0.0083	0.0034	0.0156	0.10953	
FADS2-23	rs109772589	0.0034	0.0252	0.8928	0.95942		-0.0065	0.0041	0.1139	0.26578	
C18:3n3			C18:3ntcc		
FADS1-01	rs136261927	-0.0107	0.0071	0.1317	0.29634		-0.0020	0.0020	0.3214	0.56242	
FADS1-07	rs42187261	-0.0105	0.0056	0.0610	0.29634		-0.0002	0.0016	0.9193	0.91926	
FADS1-08	rs41652284	-0.0106	0.0063	0.0914	0.29634		-0.0011	0.0018	0.5275	0.66137	
FADS2-05	rs211263660	0.0034	0.0054	0.5242	0.61156		0.0016	0.0015	0.3142	0.56242	
FADS2-14	rs211580559	0.0013	0.0052	0.8080	0.80799		0.0009	0.0015	0.5669	0.66137	
FADS2-19	rs210169303	-0.0075	0.0060	0.2117	0.29634		-0.0023	0.0017	0.1742	0.56242	
FADS2-23	rs109772589	-0.0096	0.0071	0.1757	0.29634		-0.0022	0.0020	0.2728	0.56242	
C20:3n6			C20:4n6		
FADS1-01	rs136261927	-0.0087	0.0021	0.0001	0.00041		-0.0016	0.0021	0.4558	0.53177	
FADS1-07	rs42187261	0.0037	0.0017	0.0316	0.05528		0.0042	0.0016	0.0090	0.03165	
FADS1-08	rs41652284	-0.0044	0.0020	0.0265	0.05528		-0.0024	0.0019	0.1961	0.27447	
FADS2-05	rs211263660	-0.0003	0.0017	0.8453	0.84531		-0.0026	0.0016	0.0923	0.21548	
FADS2-14	rs211580559	-0.0007	0.0016	0.6596	0.76959		-0.0023	0.0015	0.1317	0.23052	
FADS2-19	rs210169303	-0.0029	0.0019	0.1174	0.16441		0.0006	0.0017	0.7425	0.74247	
FADS2-23	rs109772589	-0.0080	0.0021	0.0002	0.00067		-0.0071	0.0020	0.0004	0.00312	
C20:5n3			C22:5n3		
FADS1-01	rs136261927	0.0001	0.0011	0.9145	0.91980		0.0035	0.0115	0.7620	0.89053	
FADS1-07	rs42187261	0.0012	0.0008	0.00358	0.03855		0.0040	0.0088	0.6489	0.89053	
FADS1-08	rs41652284	0.0008	0.0010	0.4253	0.91980		0.0051	0.0101	0.6124	0.89053	
FADS2-05	rs211263660	-0.0002	0.0008	0.8262	0.91980		-0.0018	0.0087	0.8327	0.89053	
FADS2-14	rs211580559	-0.0005	0.0008	0.5712	0.91980		0.0032	0.0086	0.7092	0.89053	
FADS2-19	rs210169303	-0.0001	0.0009	0.9198	0.91980		0.0037	0.0094	0.6914	0.89053	
FADS2-23	rs109772589	-0.0014	0.0010	0.1796	0.62855		-0.0015	0.0112	0.8905	0.89053	
Total PUFA							
FADS1-01	rs136261927	-0.0370	0.0445	0.4068	0.56947						
FADS1-07	rs42187261	-0.0026	0.0350	0.9418	0.94176						
FADS1-08	rs41652284	-0.0639	0.0396	0.1077	0.50871						
FADS2-05	rs211263660	-0.0390	0.0338	0.2498	0.50871						
FADS2-14	rs211580559	-0.0434	0.0327	0.1854	0.50871						
FADS2-19	rs210169303	0.0085	0.0375	0.8214	0.94176						
FADS2-23	rs109772589	-0.0466	0.0441	0.2907	0.50871						

Monounsaturated Fatty Acids	
C14:1			C14:1T		
FADS1-01	rs136261927	-0.0121	0.0304	0.6914	0.90775		-0.0085	0.0060	0.1575	0.25622	
FADS1-07	rs42187261	0.0369	0.0236	0.1190	0.34553		-0.0032	0.0047	0.4926	0.57135	
FADS1-08	rs41652284	-0.0018	0.0268	0.9460	0.94605		-0.0080	0.0054	0.1340	0.25622	
FADS2-05	rs211263660	-0.0332	0.0229	0.1481	0.34553		0.0092	0.0046	0.0466	0.25622	
FADS2-14	rs211580559	-0.0348	0.0223	0.1196	0.34553		0.0076	0.0045	0.0930	0.25622	
FADS2-19	rs210169303	0.0071	0.0252	0.7781	0.90775		-0.0068	0.0051	0.1830	0.25622	
FADS2-23	rs109772589	-0.0291	0.0302	0.3356	0.58729		-0.0034	0.0060	0.5713	0.57135	
C16:1			C16:1T		
FADS1-01	rs136261927	-0.0114	0.0368	0.7560	0.98916		-0.0056	0.0043	0.1929	0.45001	
FADS1-07	rs42187261	0.0004	0.0290	0.9892	0.98916		-0.0059	0.0034	0.0836	0.32095	
FADS1-08	rs41652284	-0.0344	0.0331	0.2995	0.98916		-0.0065	0.0038	0.0917	0.32095	
FADS2-05	rs211263660	-0.0193	0.0279	0.4895	0.98916		0.0030	0.0033	0.3661	0.51249	
FADS2-14	rs211580559	-0.0247	0.0273	0.3672	0.98916		0.0016	0.0032	0.6103	0.61025	
FADS2-19	rs210169303	-0.0071	0.0309	0.8181	0.98916		-0.0024	0.0036	0.5024	0.58612	
FADS2-23	rs109772589	-0.0048	0.0364	0.8957	0.98916		-0.0046	0.0042	0.2767	0.48430	
C18:1n9c			C18:1n9t		
FADS1-01	rs136261927	-0.3173	0.3567	0.3743	0.52397		0.0176	0.0207	0.3969	0.69463	
FADS1-07	rs42187261	-0.7529	0.2889	0.0095	0.06662		0.0012	0.0165	0.9413	0.94128	
FADS1-08	rs41652284	-0.2278	0.3293	0.4896	0.57125		0.0171	0.0187	0.3596	0.69463	
FADS2-05	rs211263660	0.4491	0.2696	0.0966	0.33800		-0.0043	0.0159	0.7878	0.91909	
FADS2-14	rs211580559	0.2624	0.2757	0.3417	0.52397		-0.0082	0.0154	0.5922	0.82905	
FADS2-19	rs210169303	-0.3326	0.3134	0.2892	0.52397		-0.0172	0.0179	0.3368	0.69463	
FADS2-23	rs109772589	-0.1499	0.3668	0.6830	0.68304		-0.0228	0.0208	0.2721	0.69463	
C18 :1n11t			C18:1 total		
FADS1-01	rs136261927	-0.0122	0.0494	0.8059	0.95492		-0.3779	0.3747	0.3139	0.50700	
FADS1-07	rs42187261	-0.0022	0.0390	0.9549	0.95492		-0.7697	0.3023	0.0113	0.07899	
FADS1-08	rs41652284	-0.0087	0.0432	0.8413	0.95492		-0.2703	0.3455	0.4346	0.50700	
FADS2-05	rs211263660	-0.0236	0.0376	0.5314	0.95492		0.4160	0.2842	0.1440	0.50407	
FADS2-14	rs211580559	-0.0209	0.0366	0.5673	0.95492		0.2265	0.2890	0.4336	0.50700	
FADS2-19	rs210169303	-0.0430	0.0423	0.3101	0.95492		-0.3869	0.3276	0.2384	0.50700	
FADS2-23	rs109772589	-0.0060	0.0494	0.9033	0.95492		-0.2309	0.3838	0.5479	0.54785	
Total MUFA							
FADS1-01	rs136261927	-0.6512	0.4266	0.1277	0.41320						
FADS1-07	rs42187261	-0.8044	0.3445	0.0201	0.14066						
FADS1-08	rs41652284	-0.5288	0.3911	0.1771	0.41320						
FADS2-05	rs211263660	0.3278	0.3247	0.3133	0.44377						
FADS2-14	rs211580559	0.1311	0.3264	0.6882	0.68818						
FADS2-19	rs210169303	-0.3722	0.3715	0.3170	0.44377						
FADS2-23	rs109772589	-0.1852	0.4359	0.6711	0.68818						
	Saturated Fatty Acids		
C4:0				C6:0		
FADS1-01	rs136261927	0.0232	0.0126	0.0665	0.46539		0.0167	0.0139	0.2287	0.71017	
FADS1-07	rs42187261	0.0033	0.0098	0.7390	0.86549		0.0215	0.0110	0.0504	0.35313	
FADS1-08	rs41652284	0.0138	0.0112	0.2211	0.77377		0.0112	0.0124	0.3691	0.71017	
FADS2-05	rs211263660	-0.0041	0.0095	0.6632	0.86549		-0.0086	0.0105	0.4165	0.71017	
FADS2-14	rs211580559	-0.0011	0.0093	0.9090	0.90904		-0.0009	0.0104	0.9330	0.93295	
FADS2-19	rs210169303	-0.0035	0.0105	0.7419	0.86549		0.0020	0.0116	0.8626	0.93295	
FADS2-23	rs109772589	0.0078	0.0125	0.5319	0.86549		0.0091	0.0137	0.5073	0.71017	
C8:0			C10:0		
FADS1-01	rs136261927	0.0080	0.0135	0.5561	0.91851		0.0037	0.0482	0.9389	0.96154	
FADS1-07	rs42187261	0.0246	0.0108	0.0226	0.15839		0.0872	0.0382	0.0230	0.16075	
FADS1-08	rs41652284	0.0040	0.0122	0.7428	0.91851		-0.0021	0.0439	0.9615	0.96154	
FADS2-05	rs211263660	-0.0048	0.0103	0.6411	0.91851		0.0079	0.0367	0.8304	0.96154	
FADS2-14	rs211580559	0.0028	0.0102	0.7873	0.91851		0.0284	0.0366	0.4386	0.96154	
FADS2-19	rs210169303	0.0003	0.0115	0.9824	0.98243		-0.0062	0.0410	0.8805	0.96154	
FADS2-23	rs109772589	0.0086	0.0136	0.5269	0.91851		0.0329	0.0486	0.4995	0.96154	
C11:0			C12:0		
FADS1-01	rs136261927	0.0051	0.0053	0.3365	0.47109		-0.0132	0.0669	0.8440	0.88463	
FADS1-07	rs42187261	0.0111	0.0041	0.0075	0.05265		0.1249	0.0525	0.0178	0.12467	
FADS1-08	rs41652284	0.0050	0.0047	0.2870	0.47109		-0.0098	0.0605	0.8713	0.88463	
FADS2-05	rs211263660	-0.0063	0.0040	0.1170	0.40965		0.0084	0.0509	0.8687	0.88463	
FADS2-14	rs211580559	-0.0046	0.0039	0.2407	0.47109		0.0303	0.0507	0.5502	0.88463	
FADS2-19	rs210169303	0.0010	0.0045	0.8158	0.81578		-0.0082	0.0568	0.8846	0.88463	
FADS2-23	rs109772589	-0.0020	0.0053	0.7020	0.81578		0.0278	0.0673	0.6801	0.88463	
C13:0			C14:0		
FADS1-01	rs136261927	-0.0237	0.0122	0.0520	0.35391		0.0089	0.1590	0.9552	0.95515	
FADS1-07	rs42187261	0.0053	0.0094	0.5697	0.79544		0.1719	0.1266	0.1753	0.95515	
FADS1-08	rs41652284	-0.0178	0.0108	0.1011	0.35391		-0.0219	0.1428	0.8782	0.95515	
FADS2-05	rs211263660	-0.0002	0.0093	0.9793	0.97930		0.0422	0.1204	0.7264	0.95515	
FADS2-14	rs211580559	0.0046	0.0090	0.6081	0.79544		0.0721	0.1203	0.5492	0.95515	
FADS2-19	rs210169303	0.0147	0.0104	0.1575	0.36759		-0.0924	0.1364	0.4987	0.95515	
FADS2-23	rs109772589	-0.0050	0.0121	0.6818	0.79544		0.0415	0.1610	0.7967	0.95515	
C15:0			C16:0		
FADS1-01	rs136261927	-0.0155	0.0237	0.5137	0.89900		0.6519	0.3287	0.0481	0.08447	
FADS1-07	rs42187261	0.0266	0.0181	0.1433	0.50149		0.5063	0.2555	0.0483	0.08447	
FADS1-08	rs41652284	-0.0019	0.0214	0.9306	0.93062		0.4537	0.2916	0.1206	0.14069	
FADS2-05	rs211263660	-0.0121	0.0180	0.5018	0.89900		-0.5908	0.2473	0.0173	0.07956	
FADS2-14	rs211580559	-0.0042	0.0176	0.8119	0.93062		-0.4434	0.2432	0.0691	0.09670	
FADS2-19	rs210169303	0.0321	0.0198	0.1062	0.50149		0.6281	0.2746	0.0227	0.07956	
FADS2-23	rs109772589	-0.0042	0.0236	0.8580	0.93062		0.3954	0.3224	0.2209	0.22087	
C17:0			C18:0		
FADS1-01	rs136261927	-0.0033	0.0066	0.6124	0.93749		-0.1188	0.1971	0.5470	0.69324	
FADS1-07	rs42187261	0.0010	0.0051	0.8424	0.93749		-0.1834	0.1501	0.2224	0.69324	
FADS1-08	rs41652284	0.0005	0.0058	0.9375	0.93749		0.0059	0.1687	0.9720	0.97205	
FADS2-05	rs211263660	0.0017	0.0050	0.7307	0.93749		0.1376	0.1480	0.3531	0.69324	
FADS2-14	rs211580559	0.0010	0.0048	0.8380	0.93749		0.0975	0.1448	0.5010	0.69324	
FADS2-19	rs210169303	0.0053	0.0055	0.3320	0.93749		-0.0856	0.1606	0.5942	0.69324	
FADS2-23	rs109772589	-0.0095	0.0065	0.1433	0.93749		-0.1726	0.1891	0.3619	0.69324	
C20:0			C22:0		
FADS1-01	rs136261927	-0.0005	0.0023	0.8192	0.92483		-0.0009	0.0010	0.3916	0.71670	
FADS1-07	rs42187261	0.0012	0.0018	0.4871	0.90884		0.0005	0.0008	0.5661	0.71670	
FADS1-08	rs41652284	0.0013	0.0020	0.5193	0.90884		-0.0000	0.0009	0.9613	0.96128	
FADS2-05	rs211263660	0.0002	0.0017	0.9248	0.92483		0.0008	0.0008	0.2965	0.71670	
FADS2-14	rs211580559	-0.0002	0.0017	0.9183	0.92483		0.0005	0.0008	0.4762	0.71670	
FADS2-19	rs210169303	-0.0013	0.0019	0.4900	0.90884		-0.0004	0.0009	0.6143	0.71670	
FADS2-23	rs109772589	-0.0038	0.0022	0.0888	0.62155		-0.0013	0.0010	0.1806	0.71670	
C23:0			C24:0		
FADS1-01	rs136261927	0.0001	0.0010	0.8878	0.94623		-0.0002	0.0036	0.9542	0.95416	
FADS1-07	rs42187261	0.0021	0.0008	0.0063	0.04394		0.0050	0.0029	0.0839	0.28539	
FADS1-08	rs41652284	0.0007	0.0009	0.4153	0.58135		-0.0006	0.0031	0.8527	0.95416	
FADS2-05	rs211263660	-0.0008	0.0007	0.2821	0.49370		-0.0032	0.0027	0.2379	0.39004	
FADS2-14	rs211580559	-0.0010	0.0007	0.1882	0.43908		-0.0030	0.0028	0.2786	0.39004	
FADS2-19	rs210169303	0.0001	0.0008	0.9462	0.94623		-0.0049	0.0032	0.1223	0.28539	
FADS2-23	rs109772589	-0.0015	0.0009	0.1211	0.42368		-0.0059	0.0035	0.0961	0.28539	
Total  SFA							
FADS1-01	rs136261927	0.6882	0.4397	0.1184	0.33137						
FADS1-07	rs42187261	0.8069	0.3549	0.0235	0.16482						
FADS1-08	rs41652284	0.5927	0.4028	0.1420	0.33137						
FADS2-05	rs211263660	-0.2888	0.3350	0.3891	0.54476						
FADS2-14	rs211580559	-0.0877	0.3361	0.7944	0.79436						
FADS2-19	rs210169303	0.3638	0.3827	0.3424	0.54476						
FADS2-23	rs109772589	0.2319	0.4489	0.6057	0.70668						
1Estimate = g/100g of total fat
