# Supplementary material for: Associations between variants of FADS genes and omega-3 and omega-6 milk fatty acids of Canadian Holstein cows
Source: BMC Genet. 2014 Feb 17;15:25. doi: 10.1186/1471-2156-15-25 (PMC3929906; doi:10.1186/1471-2156-15-25)
Supplement: Additional file 6: Table S6 — Pearson correlation coefficients between studied fatty acids. [file 1471-2156-15-25-S6.docx]

Table S6: Pearson correlation coefficients between studied fatty acids

| **Variable** | **C10:0** | **C11:0** | **C12:0** | **C13:0** | **C14:0** | **C14:1** | **C14:1T** | **C15:0** | **C16:0** | **C16:1** | **C16:1T** | **C17:0** | **C18:0** |
| --- | --- | --- | --- | --- | --- | --- | --- | --- | --- | --- | --- | --- | --- |
| C10:0 | 1.000 | 0.537 | 0.934 | 0.394 | 0.755 | 0.178 | -0.036 | 0.267 | 0.262 | -0.274 | -0.356 | 0.036 | -0.265 |
| C11:0 | 0.537 | 1.000 | 0.619 | 0.442 | 0.515 | 0.749 | 0.079 | 0.266 | 0.454 | 0.098 | -0.466 | 0.013 | -0.552 |
| C12:0 | 0.934 | 0.619 | 1.000 | 0.538 | 0.834 | 0.406 | 0.029 | 0.417 | 0.304 | -0.115 | -0.331 | 0.184 | -0.435 |
| C13:0 | 0.394 | 0.442 | 0.538 | 1.000 | 0.448 | 0.516 | 0.186 | 0.632 | 0.240 | 0.084 | -0.199 | 0.318 | -0.456 |
| C14:0 | 0.755 | 0.515 | 0.834 | 0.448 | 1.000 | 0.409 | 0.162 | 0.400 | 0.296 | -0.209 | -0.255 | 0.089 | -0.411 |
| C14 1 | 0.178 | 0.749 | 0.406 | 0.516 | 0.409 | 1.000 | 0.137 | 0.479 | 0.336 | 0.382 | -0.157 | 0.230 | -0.673 |
| C14:1T | -0.036 | 0.079 | 0.029 | 0.186 | 0.162 | 0.137 | 1.000 | 0.264 | -0.091 | -0.146 | 0.111 | -0.034 | 0.085 |
| C15:0 | 0.267 | 0.266 | 0.417 | 0.632 | 0.400 | 0.479 | 0.264 | 1.000 | 0.232 | 0.152 | 0.063 | 0.512 | -0.500 |
| C16:0 | 0.262 | 0.454 | 0.304 | 0.240 | 0.296 | 0.336 | -0.091 | 0.232 | 1.000 | 0.137 | -0.493 | -0.138 | -0.674 |
| C16:1 | -0.274 | 0.098 | -0.115 | 0.084 | -0.209 | 0.382 | -0.146 | 0.152 | 0.137 | 1.000 | 0.114 | 0.297 | -0.480 |
| C16:1T | -0.356 | -0.466 | -0.331 | -0.199 | -0.255 | -0.157 | 0.111 | 0.063 | -0.493 | 0.114 | 1.000 | 0.378 | 0.216 |
| C17:0 | 0.036 | 0.013 | 0.184 | 0.318 | 0.089 | 0.230 | -0.034 | 0.512 | -0.138 | 0.297 | 0.378 | 1.000 | -0.190 |
| C18:0 | -0.265 | -0.552 | -0.435 | -0.456 | -0.411 | -0.673 | 0.085 | -0.500 | -0.674 | -0.480 | 0.216 | -0.190 | 1.000 |
| C18:1total | -0.664 | -0.573 | -0.673 | -0.384 | -0.638 | -0.320 | 0.067 | -0.277 | -0.821 | 0.084 | 0.502 | 0.052 | 0.530 |
| C18:1n9c | -0.666 | -0.531 | -0.669 | -0.390 | -0.641 | -0.302 | 0.042 | -0.307 | -0.794 | 0.109 | 0.437 | 0.031 | 0.517 |
| C18:1n9t | -0.064 | -0.263 | -0.093 | 0.055 | -0.042 | -0.147 | 0.155 | -0.005 | -0.395 | -0.266 | 0.227 | -0.133 | 0.293 |
| C18:2n6CC | -0.086 | -0.143 | -0.122 | -0.122 | -0.253 | -0.126 | -0.563 | -0.282 | -0.232 | 0.062 | 0.154 | -0.008 | -0.018 |
| C18:2n6TT | 0.217 | 0.298 | 0.234 | 0.188 | 0.178 | 0.185 | 0.379 | 0.005 | 0.041 | -0.156 | -0.186 | -0.215 | -0.053 |
| C18:3tcc | -0.190 | 0.212 | -0.103 | -0.019 | -0.007 | 0.362 | 0.196 | 0.119 | 0.035 | 0.020 | 0.217 | -0.099 | -0.154 |
| C18:3n3 | -0.035 | -0.024 | -0.079 | -0.077 | -0.031 | -0.209 | 0.086 | -0.377 | -0.153 | -0.242 | -0.002 | -0.145 | 0.304 |
| C20:0 | -0.074 | -0.322 | -0.205 | -0.418 | -0.152 | -0.449 | -0.037 | -0.253 | -0.268 | -0.461 | 0.240 | -0.163 | 0.646 |
| C20:3n6 | 0.216 | 0.287 | 0.292 | 0.218 | 0.151 | 0.360 | 0.034 | 0.220 | -0.047 | -0.027 | -0.035 | 0.047 | -0.197 |
| C20:4n6 | 0.123 | 0.073 | 0.134 | 0.053 | -0.096 | 0.069 | -0.071 | 0.023 | -0.230 | 0.104 | 0.091 | 0.204 | -0.053 |
| C20:5n3 | 0.006 | -0.016 | -0.036 | -0.101 | -0.029 | -0.071 | 0.118 | -0.034 | -0.004 | -0.216 | 0.170 | -0.083 | 0.230 |
| C22:0 | 0.043 | 0.018 | -0.003 | -0.079 | 0.057 | -0.094 | 0.262 | -0.025 | 0.014 | -0.330 | 0.127 | -0.092 | 0.284 |
| C22:5n3 | 0.031 | 0.050 | 0.052 | 0.054 | 0.048 | 0.053 | 0.292 | 0.066 | -0.023 | -0.023 | 0.186 | 0.158 | -0.039 |
| C23:0 | 0.074 | 0.061 | 0.026 | -0.004 | -0.004 | -0.012 | -0.198 | -0.010 | -0.089 | -0.242 | 0.038 | -0.112 | 0.186 |
| C24:0 | 0.139 | 0.093 | 0.084 | -0.007 | 0.073 | -0.079 | 0.028 | -0.175 | 0.167 | -0.161 | 0.012 | -0.012 | 0.062 |
| C4:0 | 0.097 | 0.003 | -0.160 | -0.404 | -0.176 | -0.427 | -0.187 | -0.526 | -0.072 | -0.273 | -0.162 | -0.444 | 0.343 |
| C6:0 | 0.555 | 0.313 | 0.301 | -0.099 | 0.210 | -0.218 | -0.101 | -0.231 | 0.132 | -0.405 | -0.344 | -0.390 | 0.128 |
| C8:0 | 0.856 | 0.514 | 0.667 | 0.172 | 0.499 | -0.003 | -0.089 | -0.002 | 0.196 | -0.367 | -0.398 | -0.208 | -0.073 |
| CLA:10t12c | 0.051 | -0.091 | -0.009 | -0.153 | -0.032 | -0.153 | -0.259 | -0.031 | -0.344 | -0.253 | 0.045 | -0.007 | 0.367 |
| CLA:9c11t | -0.159 | -0.026 | -0.095 | -0.022 | 0.044 | 0.173 | 0.004 | 0.177 | -0.188 | 0.093 | 0.435 | 0.009 | -0.123 |
| MUFA | -0.659 | -0.484 | -0.617 | -0.297 | -0.594 | -0.168 | 0.070 | -0.186 | -0.789 | 0.249 | 0.543 | 0.139 | 0.391 |
| PUFA | -0.056 | -0.039 | -0.067 | -0.034 | -0.148 | -0.030 | -0.389 | -0.239 | -0.268 | -0.035 | 0.207 | -0.055 | 0.002 |
| SFA | 0.645 | 0.474 | 0.605 | 0.291 | 0.592 | 0.168 | -0.017 | 0.209 | 0.795 | -0.238 | -0.550 | -0.136 | -0.380 |
| TVA | -0.174 | -0.371 | -0.261 | -0.301 | -0.133 | -0.328 | -0.009 | -0.154 | -0.368 | -0.281 | 0.413 | -0.119 | 0.434 |

| **Variable** | **C18:1total** | **C18:1n9c** | **C18:1n9t** | **C18:2n6CC** | **C18:2n6TT** | **C18:3tcc** | **C18:3n3** | **C20:0** | **C20:3n6** | **C20:4n6** |
| --- | --- | --- | --- | --- | --- | --- | --- | --- | --- | --- |
| C10:0 | -0.664 | -0.666 | -0.064 | -0.086 | 0.217 | -0.190 | -0.035 | -0.074 | 0.216 | 0.123 |
| C11:0 | -0.573 | -0.531 | -0.263 | -0.143 | 0.298 | 0.212 | -0.024 | -0.322 | 0.287 | 0.073 |
| C12:0 | -0.673 | -0.669 | -0.093 | -0.122 | 0.234 | -0.103 | -0.079 | -0.205 | 0.292 | 0.134 |
| C13:0 | -0.384 | -0.390 | 0.055 | -0.122 | 0.188 | -0.019 | -0.077 | -0.418 | 0.218 | 0.053 |
| C14:0 | -0.638 | -0.641 | -0.042 | -0.253 | 0.178 | -0.007 | -0.031 | -0.152 | 0.151 | -0.096 |
| C14:1 | -0.320 | -0.302 | -0.147 | -0.126 | 0.185 | 0.362 | -0.209 | -0.449 | 0.360 | 0.069 |
| C14:1T | 0.067 | 0.042 | 0.155 | -0.563 | 0.379 | 0.196 | 0.086 | -0.037 | 0.034 | -0.071 |
| C15:0 | -0.277 | -0.307 | -0.005 | -0.282 | 0.005 | 0.119 | -0.377 | -0.253 | 0.220 | 0.023 |
| C16:0 | -0.821 | -0.794 | -0.395 | -0.232 | 0.041 | 0.035 | -0.153 | -0.268 | -0.047 | -0.230 |
| C16:1 | 0.084 | 0.109 | -0.266 | 0.062 | -0.156 | 0.020 | -0.242 | -0.461 | -0.027 | 0.104 |
| C16:1T | 0.502 | 0.437 | 0.227 | 0.154 | -0.186 | 0.217 | -0.002 | 0.240 | -0.035 | 0.091 |
| C17:0 | 0.052 | 0.031 | -0.133 | -0.008 | -0.215 | -0.099 | -0.145 | -0.163 | 0.047 | 0.204 |
| C18:0 | 0.530 | 0.517 | 0.293 | -0.018 | -0.053 | -0.154 | 0.304 | 0.646 | -0.197 | -0.053 |
| C18:1total | 1.000 | 0.983 | 0.290 | 0.249 | -0.120 | 0.098 | 0.009 | 0.132 | -0.029 | 0.183 |
| C18:1n9c | 0.983 | 1.000 | 0.236 | 0.237 | -0.117 | 0.105 | 0.002 | 0.107 | -0.030 | 0.187 |
| C18:1n9t | 0.290 | 0.236 | 1.000 | 0.087 | 0.173 | -0.120 | 0.037 | 0.028 | 0.023 | 0.087 |
| C18:2n6CC | 0.249 | 0.237 | 0.087 | 1.000 | -0.057 | -0.052 | 0.138 | -0.071 | 0.267 | 0.453 |
| C18:2n6TT | -0.120 | -0.117 | 0.173 | -0.057 | 1.000 | 0.082 | 0.126 | -0.214 | 0.329 | 0.199 |
| C18:3tcc | 0.098 | 0.105 | -0.120 | -0.052 | 0.082 | 1.000 | 0.062 | 0.314 | 0.175 | -0.107 |
| C18:3n3 | 0.009 | 0.002 | 0.037 | 0.138 | 0.126 | 0.062 | 1.000 | 0.292 | -0.198 | -0.207 |
| C20:0 | 0.132 | 0.107 | 0.028 | -0.071 | -0.214 | 0.314 | 0.292 | 1.000 | -0.159 | -0.235 |
| C20:3n6 | -0.029 | -0.030 | 0.023 | 0.267 | 0.329 | 0.175 | -0.198 | -0.159 | 1.000 | 0.562 |
| C20:4n6 | 0.183 | 0.187 | 0.087 | 0.453 | 0.199 | -0.107 | -0.207 | -0.235 | 0.562 | 1.000 |
| C20:5n3 | -0.076 | -0.079 | -0.098 | -0.143 | 0.057 | 0.504 | 0.305 | 0.636 | -0.041 | -0.183 |
| C22:0 | -0.130 | -0.151 | -0.093 | -0.276 | 0.067 | 0.528 | 0.417 | 0.704 | -0.093 | -0.262 |
| C22:5n3 | -0.003 | -0.032 | 0.005 | -0.064 | 0.203 | 0.168 | 0.231 | 0.034 | 0.095 | 0.219 |
| C23:0 | -0.038 | -0.030 | 0.062 | 0.179 | -0.127 | 0.320 | 0.238 | 0.479 | 0.145 | -0.013 |
| C24:0 | -0.237 | -0.247 | -0.178 | -0.034 | 0.079 | 0.216 | 0.512 | 0.305 | -0.052 | -0.155 |
| C4:0 | -0.001 | 0.015 | -0.015 | 0.102 | -0.010 | -0.177 | 0.215 | 0.173 | -0.210 | -0.031 |
| C6:0 | -0.360 | -0.352 | -0.037 | -0.023 | 0.114 | -0.195 | 0.147 | 0.153 | -0.046 | 0.015 |
| C8:0 | -0.539 | -0.534 | -0.066 | -0.039 | 0.198 | -0.182 | 0.071 | 0.033 | 0.109 | 0.087 |
| CLA:10t12c | 0.168 | 0.183 | 0.083 | 0.098 | -0.286 | 0.054 | 0.106 | 0.488 | 0.064 | -0.007 |
| CLA:9c11t | 0.188 | 0.140 | 0.209 | 0.182 | -0.128 | 0.403 | 0.079 | 0.140 | 0.074 | -0.054 |
| MUFA | 0.959 | 0.951 | 0.277 | 0.270 | -0.106 | 0.134 | -0.027 | 0.021 | 0.023 | 0.223 |
| PUFA | 0.231 | 0.207 | 0.168 | 0.843 | 0.084 | 0.109 | 0.398 | 0.005 | 0.295 | 0.353 |
| SFA | -0.956 | -0.945 | -0.283 | -0.353 | 0.102 | -0.140 | -0.016 | -0.020 | -0.047 | -0.249 |
| TVA | 0.268 | 0.179 | 0.220 | 0.119 | -0.204 | 0.128 | 0.257 | 0.545 | -0.146 | -0.201 |

| **Variable** | **C20:5n3** | **C22:0** | **C22:5n3** | **C23:0** | **C24:0** | **C4:0** | **C6:0** | **C8:0** | **CLA:10t12c** | **CLA:9c11t** | **MUFA** | **PUFA** | **SFA** |
| --- | --- | --- | --- | --- | --- | --- | --- | --- | --- | --- | --- | --- | --- |
| C10:0 | 0.006 | 0.043 | 0.031 | 0.074 | 0.139 | 0.097 | 0.555 | 0.856 | 0.051 | -0.159 | -0.659 | -0.056 | 0.645 |
| C11:0 | -0.016 | 0.018 | 0.050 | 0.061 | 0.093 | 0.003 | 0.313 | 0.514 | -0.091 | -0.026 | -0.484 | -0.039 | 0.474 |
| C12:0 | -0.036 | -0.003 | 0.052 | 0.026 | 0.084 | -0.160 | 0.301 | 0.667 | -0.009 | -0.095 | -0.617 | -0.067 | 0.605 |
| C13:0 | -0.101 | -0.079 | 0.054 | -0.004 | -0.007 | -0.404 | -0.099 | 0.172 | -0.153 | -0.022 | -0.297 | -0.034 | 0.291 |
| C14:0 | -0.029 | 0.057 | 0.048 | -0.004 | 0.073 | -0.176 | 0.210 | 0.499 | -0.032 | 0.044 | -0.594 | -0.148 | 0.592 |
| C14:1 | -0.071 | -0.094 | 0.053 | -0.012 | -0.079 | -0.427 | -0.218 | -0.003 | -0.153 | 0.173 | -0.168 | -0.030 | 0.168 |
| C14:1T | 0.118 | 0.262 | 0.292 | -0.198 | 0.028 | -0.187 | -0.101 | -0.089 | -0.259 | 0.004 | 0.070 | -0.389 | -0.017 |
| C15:0 | -0.034 | -0.025 | 0.066 | -0.010 | -0.175 | -0.526 | -0.231 | -0.002 | -0.031 | 0.177 | -0.186 | -0.239 | 0.209 |
| C16:0 | -0.004 | 0.014 | -0.023 | -0.089 | 0.167 | -0.072 | 0.132 | 0.196 | -0.344 | -0.188 | -0.789 | -0.268 | 0.795 |
| C16:1 | -0.216 | -0.330 | -0.023 | -0.242 | -0.161 | -0.273 | -0.405 | -0.367 | -0.253 | 0.093 | 0.249 | -0.035 | -0.238 |
| C16:1T | 0.170 | 0.127 | 0.186 | 0.038 | 0.012 | -0.162 | -0.344 | -0.398 | 0.045 | 0.435 | 0.543 | 0.207 | -0.550 |
| C17:0 | -0.083 | -0.092 | 0.158 | -0.112 | -0.012 | -0.444 | -0.390 | -0.208 | -0.007 | 0.009 | 0.139 | -0.055 | -0.136 |
| C18:0 | 0.230 | 0.284 | -0.039 | 0.186 | 0.062 | 0.343 | 0.128 | -0.073 | 0.367 | -0.123 | 0.391 | 0.002 | -0.380 |
| C18:1total | -0.076 | -0.130 | -0.003 | -0.038 | -0.237 | -0.001 | -0.360 | -0.539 | 0.168 | 0.188 | 0.959 | 0.231 | -0.956 |
| C18:1n9c | -0.079 | -0.151 | -0.032 | -0.030 | -0.247 | 0.015 | -0.352 | -0.534 | 0.183 | 0.140 | 0.951 | 0.207 | -0.945 |
| C18:1n9t | -0.098 | -0.093 | 0.005 | 0.062 | -0.178 | -0.015 | -0.037 | -0.066 | 0.083 | 0.209 | 0.277 | 0.168 | -0.283 |
| C18:2n6CC | -0.143 | -0.276 | -0.064 | 0.179 | -0.034 | 0.102 | -0.023 | -0.039 | 0.098 | 0.182 | 0.270 | 0.843 | -0.353 |
| C18:2n6TT | 0.057 | 0.067 | 0.203 | -0.127 | 0.079 | -0.010 | 0.114 | 0.198 | -0.286 | -0.128 | -0.106 | 0.084 | 0.102 |
| C18:3tcc | 0.504 | 0.528 | 0.168 | 0.320 | 0.216 | -0.177 | -0.195 | -0.182 | 0.054 | 0.403 | 0.134 | 0.109 | -0.140 |
| C18:3n3 | 0.305 | 0.417 | 0.231 | 0.238 | 0.512 | 0.215 | 0.147 | 0.071 | 0.106 | 0.079 | -0.027 | 0.398 | -0.016 |
| C20:0 | 0.636 | 0.704 | 0.034 | 0.479 | 0.305 | 0.173 | 0.153 | 0.033 | 0.488 | 0.140 | 0.021 | 0.005 | -0.020 |
| C20:3n6 | -0.041 | -0.093 | 0.095 | 0.145 | -0.052 | -0.210 | -0.046 | 0.109 | 0.064 | 0.074 | 0.023 | 0.295 | -0.047 |
| C20:4n6 | -0.183 | -0.262 | 0.219 | -0.013 | -0.155 | -0.031 | 0.015 | 0.087 | -0.007 | -0.054 | 0.223 | 0.353 | -0.249 |
| C20:5n3 | 1.000 | 0.842 | 0.289 | 0.459 | 0.446 | 0.053 | 0.089 | 0.050 | 0.189 | 0.138 | -0.112 | 0.027 | 0.110 |
| C22:0 | 0.842 | 1.000 | 0.314 | 0.417 | 0.472 | 0.048 | 0.118 | 0.087 | 0.211 | 0.133 | -0.185 | -0.066 | 0.188 |
| C22:5n3 | 0.289 | 0.314 | 1.000 | 0.022 | 0.276 | -0.077 | 0.016 | 0.031 | -0.163 | 0.071 | 0.020 | 0.082 | -0.024 |
| C23:0 | 0.459 | 0.417 | 0.022 | 1.000 | 0.165 | 0.090 | 0.148 | 0.135 | 0.432 | 0.296 | -0.054 | 0.290 | 0.021 |
| C24:0 | 0.446 | 0.472 | 0.276 | 0.165 | 1.000 | 0.172 | 0.225 | 0.191 | -0.084 | -0.078 | -0.279 | 0.158 | 0.256 |
| C4:0 | 0.053 | 0.048 | -0.077 | 0.090 | 0.172 | 1.000 | 0.790 | 0.500 | 0.067 | -0.176 | -0.088 | 0.074 | 0.078 |
| C6:0 | 0.089 | 0.118 | 0.016 | 0.148 | 0.225 | 0.790 | 1.000 | 0.878 | 0.135 | -0.204 | -0.437 | -0.016 | 0.431 |
| C8:0 | 0.050 | 0.087 | 0.031 | 0.135 | 0.191 | 0.500 | 0.878 | 1.000 | 0.108 | -0.189 | -0.577 | -0.013 | 0.563 |
| CLA:10t12c | 0.189 | 0.211 | -0.163 | 0.432 | -0.084 | 0.067 | 0.135 | 0.108 | 1.000 | 0.241 | 0.130 | 0.132 | -0.134 |
| CLA:9c11t | 0.138 | 0.133 | 0.071 | 0.296 | -0.078 | -0.176 | -0.204 | -0.189 | 0.241 | 1.000 | 0.260 | 0.396 | -0.291 |
| MUFA | -0.112 | -0.185 | 0.020 | -0.054 | -0.279 | -0.088 | -0.437 | -0.577 | 0.130 | 0.260 | 1.000 | 0.234 | -0.990 |
| PUFA | 0.027 | -0.066 | 0.082 | 0.290 | 0.158 | 0.074 | -0.016 | -0.013 | 0.132 | 0.396 | 0.234 | 1.000 | -0.343 |
| SFA | 0.110 | 0.188 | -0.024 | 0.021 | 0.256 | 0.078 | 0.431 | 0.563 | -0.134 | -0.291 | -0.990 | -0.343 | 1.000 |
| TVA | 0.278 | 0.316 | -0.016 | 0.281 | 0.101 | 0.148 | 0.031 | -0.082 | 0.314 | 0.557 | 0.204 | 0.259 | -0.222 |

| **Variable** | **TVA** | C10:0 to C16:0 | C10:0 to C16:0 | C10:0 to C16:0 |
| --- | --- | --- | --- | --- |
| C10:0 | -0.174 | 0.565 | -0.262 | 0.541 |
| C11:0 | -0.371 | 0.572 | -0.551 | 0.302 |
| C12:0 | -0.261 | 0.624 | -0.432 | 0.290 |
| C13:0 | -0.301 | 0.405 | -0.455 | -0.115 |
| C14:0 | -0.133 | 0.613 | -0.409 | 0.195 |
| C14:1 | -0.328 | 0.410 | -0.673 | -0.228 |
| C14:1T | -0.009 | -0.050 | 0.083 | -0.124 |
| C15:0 | -0.154 | 0.373 | -0.497 | -0.268 |
| C16:0 | -0.368 | 0.899 | -0.672 | 0.096 |
| C16:1 | -0.281 | 0.049 | -0.482 | -0.394 |
| C16:1T | 0.413 | -0.496 | 0.219 | -0.322 |
| C17:0 | -0.119 | 0.006 | -0.187 | -0.384 |
| C18:0 | 0.434 | -0.701 | 1.000 | 0.139 |
| C18:1total | 0.268 | -0.944 | 0.525 | -0.328 |
| C18:1n9c | 0.179 | -0.927 | 0.511 | -0.318 |
| C18:1n9t | 0.220 | -0.336 | 0.288 | -0.031 |
| C18:2n6CC | 0.119 | -0.269 | -0.018 | 0.014 |
| C18:2n6TT | -0.204 | 0.084 | -0.058 | 0.115 |
| C18:3tcc | 0.128 | -0.048 | -0.142 | -0.193 |
| C18:3n3 | 0.257 | -0.150 | 0.310 | 0.166 |
| C20:0 | 0.545 | -0.284 | 0.659 | 0.139 |
| C20:3n6 | -0.146 | 0.035 | -0.198 | -0.043 |
| C20:4n6 | -0.201 | -0.177 | -0.057 | 0.032 |
| C20:5n3 | 0.278 | -0.034 | 0.245 | 0.082 |
| C22:0 | 0.316 | 0.011 | 0.300 | 0.100 |
| C22:5n3 | -0.016 | -0.018 | -0.033 | 0.004 |
| C23:0 | 0.281 | -0.083 | 0.198 | 0.150 |
| C24:0 | 0.101 | 0.161 | 0.069 | 0.228 |
| C4:0 | 0.148 | -0.129 | 0.341 | 0.832 |
| C6:0 | 0.031 | 0.209 | 0.129 | 0.990 |
| C8:0 | -0.082 | 0.396 | -0.071 | 0.866 |
| CLA:10t12c | 0.314 | -0.267 | 0.374 | 0.124 |
| CLA:9c11t | 0.557 | -0.156 | -0.117 | -0.193 |
| MUFA | 0.204 | -0.903 | 0.386 | -0.402 |
| PUFA | 0.259 | -0.268 | 0.004 | 0.026 |
| SFA | -0.222 | 0.904 | -0.375 | 0.391 |
| TVA | 1.000 | -0.348 | 0.441 | 0.042 |
| C10:0 to C16:0 | -0.348 | 1.000 | -0.698 | 0.175 |
| C18:0 to C24:0 | 0.441 | -0.698 | 1.000 | 0.139 |
| C4:0 to C8:0 | 0.042 | 0.175 | 0.139 | 1.000 |
